# Dissipation study of ten insecticides in apples under field conditions

**DOI:** 10.1002/jsfa.14370

**Published:** 2025-05-12

**Authors:** Dana Schusterova, Jitka Stara, Frantisek Kocourek, Vojtech Hrbek, Petr Mraz, Vit Kosek, Petra Vackova, Vladimir Kocourek, Jana Hajslova, Tereza Horska

**Affiliations:** ^1^ Department of Food Analysis and Nutrition Faculty of Food and Biochemical Technology, University of Chemistry and Technology Prague Czech Republic; ^2^ Division of Crop Protection and Plant Health Czech Agrifood Research Center Prague Czech Republic

**Keywords:** apples, field trials, insecticides, pesticide dissipation, QuEChERS

## Abstract

**BACKGROUND:**

Apples are among the most widely cultivated fruits in temperate climate zone. Given the potential of pests and diseases to cause significant damage to fruit production, orchardists rely on using insecticides and fungicides in infested orchards to protect apple yields. In this study, the dissipation of ten insecticides was monitored under field conditions in two apple varieties (Rosana and Selena) in an apple orchard. Pesticide residues in apples were determined using the QuEChERS extraction method followed by ultrahigh‐performance liquid chromatography coupled to tandem mass spectrometry.

**RESULTS:**

The dissipation rate of all insecticides applied more than 80 days before harvest followed first‐order kinetics. Mean dissipation half‐lives were calculated ranging from 4.5 days (spinosad) to 66.4 days (flonicamid, sum in accordance with residue definition). At the end of the preharvest interval, all analytes tested were found at concentrations below 30% of the established maximum residue levels, except flonicamid (sum) and pirimicarb. As metabolites of flonicamid and spirotetramat (i.e., TFNA, TFNG and spirotetramat‐enol) were included in the residue definition, changes in their levels were also monitored. In the case of flupyradifurone, a statistically significant difference in dissipation rate between apple varieties was found.

**CONCLUSION:**

The results provided a better understanding of the fate of insecticides in apples in apple orchards and thus the potential risks associated with their use. The results of this study also provide a scientific basis for the appropriate selection and use of pesticides for the integrated pest management practices in apple orchards. © 2025 The Author(s). *Journal of the Science of Food and Agriculture* published by John Wiley & Sons Ltd on behalf of Society of Chemical Industry.

## INTRODUCTION

Apples (*Malus domestica*) are one of the most widely cultivated fruits in temperate climate zone, ranking third in world fruit production after tomatoes and bananas. In 2022, the world production of apples was almost 96 million tons, approximately half of which was produced in China.^1^, ^2^ In the Czech Republic, apple orchards cover an area of about 4000 ha with the production of more than 36 thousand tons.^3^ In the long term, the consumption of apples may have a positive effect on some chronic diseases (e.g., obesity, diabetes, cancer, or cardiovascular diseases). In addition to the high content of soluble fiber, the beneficial bioactivities of apples are associated with a wide range of secondary metabolites such as vitamins (e.g., vitamins C, B_6_ and E) and polyphenols (e.g., flavanols, flavonols, dihydrochalcones and anthocyanins). The profiles of these bioactive components are quite variable, depending on the apple varieties, their geographical origin and also on the preharvest and postharvest agriculture practices used. It should be noted that some differences in the composition of apples can even be found between certain parts of the fruit.^4^, ^5^ Apples are an important source of these health‐promoting compounds for Czech consumers, as they are the most consumed fruit in the Czech Republic with a *per capita* consumption of 25.7 kg (representing around 30% of total fruit consumed).^6^

Besides the consumer demand for high sensory quality, the occurrence of pesticide residues is also of concern. Therefore, in order to limit the use of synthetic pesticides as much as possible, a carefully designed strategy based on integrated pest management (IPM) should be applied.^7^ The effectiveness of IPM is clearly associated with a deep understanding of possible apple tree pests and diseases and knowledge of their life cycles. The codling moth (*Cydia pomonella*) is a major pest of pome fruit. Its larvae bore into the center of the fruit, stop growth and cause premature ripening. Because this insect can severely damage fruit production, apple‐producing countries rely on the frequent use of insecticides, such as acetamiprid, spinosad or chlorantraniliprole, in infested orchards to protect apple tree yields.^8^ However, the increasing frequency of insecticide treatments globally has led to pest resistance to many of the recommended pesticides belonging to different chemical groups^8^, ^9^, ^10^ even to biocontrol agent *C. pomonella* granulovirus.^11^, ^12^, ^13^

To reduce the number of eggs laid in apple buds, insecticide treatment against the adult apple blossom weevil (*Anthonomus pomorum*) commonly begins in early spring. Its larvae nip the buds, and the damaged blossoms then fail to develop into fruit, or the fruit is malformed. By using more selective insecticides instead of broad‐spectrum ones, blossom weevil populations can increase to damaging levels. This is particularly problematic in years with high population densities and/or low bud set.^14^ This insect species can be effectively controlled with insecticides such as spinosad.^1^, ^15^, ^16^ Insecticide treatments in apple orchards should also target other important insect pests such as eggs and larvae of leafroller moths (*Tortricidae*), adults and hatching larvae of apple sawfly (*Hoplocampa testudinea*) and armored scale insects (*Diaspididae*).^16^, ^17^, ^18^ Various species of aphids such as rosy apple aphid (*Dysaphis plantaginea*), green apple aphid (*Aphis pomi*) or woolly apple aphid (*Eriosoma lanigerum*) cause damage to different parts of apple trees. Insecticide treatments against them are in place throughout the apple growing season.^19^, ^20^, ^21^

As pesticide residues may pose risks to human health and also threaten non‐target organisms, understanding the fate of pesticides is necessary to ensure food safety and environmental protection. Pesticide dissipation is mostly described in experimental studies by measuring the concentration of pesticide residues in plants over time and estimating process‐specific rate coefficients or dissipation half‐lives (*t*_1/2_).^22^ However, assessing pesticide dissipation in apple orchards is very challenging due to several factors, including a wide range of physico‐chemical properties of pesticide preparations, variations in residue concentrations in an apple canopy, morphological characteristics of apple trees or the important role weather conditions play in a particular locality.^5^, ^23^ Pesticide dissipation rate in apples has been investigated in several previously published studies; acetamiprid,^24^, ^25^ chlorantraniliprole,^26^, ^27^ chlorpyrifos,^24^ chlorpyrifos‐methyl,^26^ imidacloprid,^23^ indoxacarb^26^ or pirimicarb^5^, ^27^ were among the insecticides whose fate was studied. In addition to the insecticides mentioned, other pesticide ingredients (e.g., cyantraniliprole, flupyradifurone, pyriproxyfen or tebufenozide) are registered for use on apples. However, knowledge of their fate after treatment is limited. In any case, consistent adherence on preharvest intervals (PHIs) is critical for safe apple production to ensure that pesticide residue levels in the crops fall below established maximum residue levels (MRLs).^28^

The objectives of this study were to evaluate the dissipation kinetic parameters and dissipation half‐lives of ten insecticides in treated apples and to monitor the formation and changes in the levels of selected metabolites of flonicamid and spirotetramat. The selected insecticides evaluated belong to the basic elements of chemical pest control in Central Europe. This study also aimed at the comparison of pesticide dissipation rates in two locally grown apple varieties (Rosana and Selena).

## METHODS AND MATERIALS

### Reagents and materials

Methanol (LC–MS grade), acetonitrile (HPLC grade), formic acid (99%), ammonium acetate (LC–MS grade), and ammonium formate (LC–MS grade) were supplied by Merck KGaA (Darmstadt, Germany). Sodium chloride (p.a.) was purchased from Lach‐ner Ltd (Neratovice, Czech Republic). Acetone (p.a.) was supplied by Penta (Chrudim, Czech Republic). Anhydrous magnesium sulfate (p.a.) was obtained from Honeywell Fluka™ (Charlotte, NC, USA). Purified water (TOC ≤ 5 μg L^−1^) was prepared using a Millipore Milli‐Q system (Millipore, Bedford, MA, USA).

Certified analytical standards of insecticides (acetamiprid, chlorantraniliprole, cyantraniliprole, flonicamid, flupyradifurone, pirimicarb, pyriproxyfen, spinosad (spinosyn A and spinosyn D), spirotetramat and tebufenozide), selected pesticide metabolites (flonicamid metabolite TFNA, flonicamid metabolite TFNG, spirotetramat‐enol, spirotetramat‐enol glucoside, spirotetramat‐ketohydroxy and spirotetramat‐monohydroxy) and internal standards (triphenyl phosphate and nicarbazin) were purchased from Dr Ehrenstorfer GmbH (Augsburg, Germany), HPC Standards GmbH (Cunnersdorf, Germany), Honeywell Fluka (Charlotte, NC, USA) and Sigma‐Aldrich (Darmstadt, Germany). The purity of the standards was in the range of 96.0–99.9%.

Plant protection products (PPPs) were supplied by Adama Ltd (Tel Aviv, Israel), Bayer AG (Monheim am Rhein, Germany), Corteva Agriscience™ (Indianapolis, IN, USA), FMC Agro Czech Republic Ltd (Prague, Czech Republic), Galenika‐Fitofarmacija A.D. (Zemun, Belgrade), ISK Biosciences Corporation (Concord Township, OH, USA) and Nisso Chemical Europe GmbH (Duesseldorf, Germany). The PPPs used in these field trials are summarized in Table 1, the list of authorized uses of tested PPPs for apple trees in the Czech Republic is shown in Table S1 in the Supporting Information.

**Table 1:** Plant protection products (PPPs) and their application doses

Trade name of PPP (pesticide formulation)	Active ingredient (content of active ingredient in PPP)	Dose of PPP^a^ (kg ha^−1^)	Dose of active ingredient (kg ha^−1^)
Benevia/Exirel (oil dispersion/suspo‐emulsion)	Cyantraniliprole (100 g L^−1^)	0.60	0.06
Coragen 20 SC (suspension concentrate)	Chlorantraniliprole (200 g L^−1^)	0.16	0.03
Harpun (emulsifiable concentrate)	Pyriproxyfen (100 g L^−1^)	1.00	0.10
Mimic (suspension concentrate)	Tebufenozide (240 g L^−1^)	0.75	0.18
Mospilan 20 SP (soluble powder)	Acetamiprid (200 g kg^−1^)	0.25	0.05
Movento® 100 SC (suspension concentrate)	Spirotetramat (100 g L^−1^)	2.25	0.23
Pirimor 50 WG (water‐dispersible granules)	Pirimicarb (500 g kg^−1^)	0.50	0.25
Sivanto Prime (soluble concentrate)	Flupyradifurone (200 g L^−1^)	0.60	0.12
SpinTor (suspension concentrate)	Spinosad (240 g L^−1^)	0.60	0.14
Teppeki (water‐dispersible granules)	Flonicamid (500 g kg^−1^)	0.14	0.07

^a^ Application doses were in accordance with the recommendations of the manufacturers of the individual PPPs, which were applied in the orchard diluted in 500 L of water.

### Field trials

**Figure 1:**
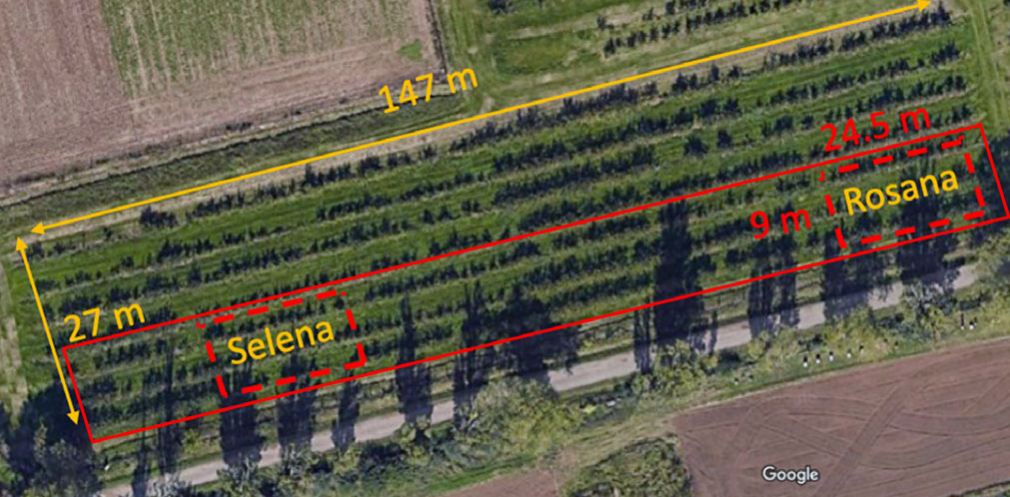
Treated area (red rectangle) and locations of apple varieties Rosana and Selena in the apple orchard.

The supervised field trials were conducted from 2020 to 2023 in the experimental apple orchard of the Czech Agrifood Research Center (formerly Crop Research Institute) in Prague, Czech Republic (location: 50.086965 N, 14.298240 E). Treated area in the apple orchard is shown in Fig. 1. The apple trees in the orchard of the ‘Rosana’ and ‘Selena’ varieties were planted in 1992 in rows 4.5 m apart, with a distance of 1.7 m between the trees within the rows.

**Figure 2:**
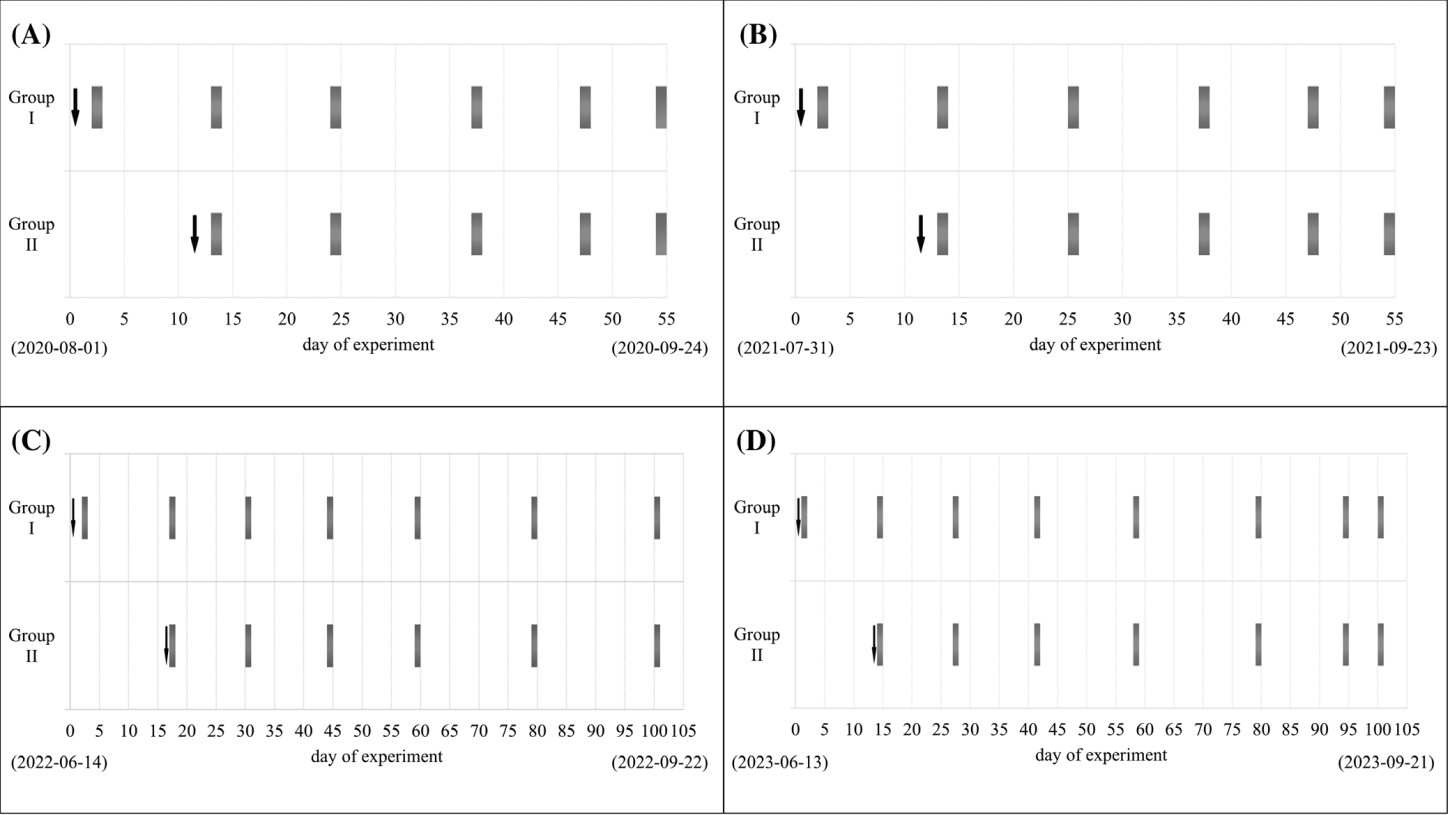
Timetable of pesticide treatments (black arrow) and sampling (gray boxes) of apples in 2020 (A), 2021 (B), 2022 (C) and 2023 (D).

Apple trees of both varieties were treated with insecticidal PPPs according to the program summarized in Fig. 2. In each year of experimental treatment, the insecticides were divided into two groups (i.e., Group I and Group II) that differed in the date of their use for treatment. Detailed information (e.g., classification of insecticides into groups) can be found in Table S2. Application doses (see Table 1) of insecticides were used in accordance with the recommendations of the manufacturers of individual PPP.^29^ Aqueous suspensions of the insecticides were prepared by diluting PPPs with water (500 L ha^−1^). In 2020 and 2021, the pesticide treatments were carried out in the period from the end of July to mid‐August. However, in 2022 and 2023, insecticide preparations were sprayed in the period from mid‐June to the end of June. The application of the pesticide preparations was carried out with a HARDI lift‐mounted sprayer (Exel Industries S.A., Paris, France). Weather conditions during the 4 years of field trials in the apple orchard are summarized in Figs S1 **–**S4 in the Supporting Information. The agro‐meteorological station is located about 200 m from the experimental apple orchard of the Czech Agrifood Research Center in Prague (location: 50.085000 N, 14.298333 E).

### Samples

Samples of both apple varieties were collected according to the program shown in Fig. 2. Developing fruits were sampled 1 or 3 days after treatment with each group of PPPs and then at intervals of 1 to 3 weeks until harvest. Samples of apples were collected from different parts of the canopy of randomly selected apple trees in the treated section of the apple orchard. Three samples of fruit were collected each time, each sample consisting of fruit weighing approximately 1 kg. At the time of sampling, individual samples of apples were placed separately in plastic bags and transported in cooled boxes to the laboratory for analysis. After sample reduction by quartering, the composite laboratory apple samples were cut into small pieces, deep frozen (−20 °C) for 12 h, and then homogenized using a laboratory blender. Samples were analyzed immediately after homogenization or stored deeply frozen and processed within 3 days. The stability of selected insecticides in the comminuted apples was checked in the stability study carried out on spiked apple homogenates.

### Sample preparation

The extracts of apples were prepared according to the QuEChERS extraction procedure.^30^ The homogenized sample (10 g) was weighed into a 50 mL centrifuge tube and acetonitrile (10 mL) was added. After the shaking of the tube on a vertical laboratory shaker (1000 strokes min^−1^, 2 min), a mixture of sodium chloride (1 g) and magnesium sulfate (4 g) was added, and the sample was shaken for 1 min. Then, an internal standard (100 μL; 5000 ng mL^−1^) was added and the tube was centrifuged (5 min, 13 000 × *g*). An aliquot of the extract was transferred to a vial for liquid chromatography coupled to tandem mass spectrometry (LC–MS/MS) analysis.

An acidified version of the QuEChERS extraction method was used in the case of analysis of metabolites of flonicamid (TFNA and TFNG). The apple samples were extracted using acetonitrile containing 1% (*v/v*) formic acid. The next steps of sample preparation followed the procedure described in the previous paragraph.^31^

### Analytical standards preparation

Solid analytical standards of pesticides were dissolved (based on the solubility of each compound) in acetonitrile or methanol containing 1% (*v/v*) formic acid. Acidified acetonitrile was used for the following standard dilutions. Individual stock standard solutions were mixed in two separate mixtures: MIX A containing parent pesticides and MIX B containing targeted metabolites (50 000 ng mL^−1^). These mixtures were then used to prepare working standard solutions at 20, 40, 100, 200, 400, 1000 and 2000 ng mL^−1^. All prepared stock pesticide solutions were stored in volumetric flasks in a freezer (−20 °C) protected from light.

To compensate matrix effects, the quantitative analysis of pesticide residues in apples was performed using a matrix‐matched calibration. Matrix calibration standards (separately for MIX A and MIX B) were prepared by diluting working standard solutions (50 μL, 20–2000 ng mL^−1^) and internal standard solution (50 μL, 1000 ng mL^−1^) with QuEChERS extract of blank sample of the respective apple variety (900 μL). The blank apple extract was prepared according to the procedure described in the section ‘Sample preparation’.

### LC–MS/MS analysis of pesticide residues

Analyses of 16 insecticides and their relevant metabolites in apples were performed using an Agilent 1290 Infinity II liquid chromatograph coupled to a Triple Quadrupole G6495C mass spectrometer (both Agilent Technologies, Santa Clara, CA, USA). A reversed‐phase ACQUITY HSS T3 UPLC column (100 mm × 2.1 mm, 1.8 μm; Waters Corporation, Milford, MA, USA) was employed for sample separation. The injection volume was 2 μL. Mass detection was performed by electrospray ionization (ESI, both ionization modes) in dynamic multiple reaction monitoring (MRM) mode. MassHunter Workstation software (version 10.0; Agilent Technologies) was used for data acquisition. The measured MRM transitions, retention time and collision energies for the target analytes are given in Table 2. Detailed parameters of the LC–MS/MS validated method (i.e., ultrahigh‐performance liquid chromatography (UHPLC) gradient and ESI source parameters) were described in our previous study.^32^

**Table 2:** Liquid chromatography coupled to tandem mass spectrometry (LC–MS/MS) measurement conditions for the targeted analytes

Pesticide/metabolite	ESI^a^ mode	RT^b^ (min)	MS/MS transitions (collision energy (V))
Acetamiprid	ESI+	4.1	223.1 > 126.1 (28); 223.1 > 56.0 (16)
Chlorantraniliprole	ESI+	7.2	483.9 > 452.9 (20); 483.9 > 285.9 (12)
Cyantraniliprole	ESI+	5.9	473.0 > 284.0 (12); 473.0 > 177.0 (52)
Flonicamid	ESI+	3.6	230.1 > 203.0 (16); 230.1 > 98.0 (48)
Flonicamid metabolite: TFNA	ESI−	2.6	190.0 > 126.0 (24); 190.0 > 69.0 (36)
Flonicamid metabolite: TFNG	ESI−	3.0	247.0 > 182.9 (12); 247.0 > 163.0 (20)
Flupyradifurone	ESI+	4.0	289.1 > 126.0 (24); 289.1 > 99.0 (60)
Pirimicarb	ESI+	5.4	239.1 > 182.2 (12); 239.1 > 72.1 (24)
Pyriproxyfen	ESI+	11.4	322.2 > 185.1 (24); 322.2 > 96.0 (24)
Spinosyn A	ESI+	10.1	732.6 > 142.2 (28); 732.6 > 98.2 (52)
Spinosyn D	ESI+	10.5	746.5 > 142.1 (36); 746.5 > 98.5 (52)
Spirotetramat	ESI+	8.7	374.2 > 302.2 (16); 374.2 > 216.1 (36)
Spirotetramat‐enol	ESI+	6.2	302.0 > 216.0 (28); 302.0 > 117.0 (52)
Spirotetramat‐enol glucoside	ESI+	3.7	464.0 > 270.0 (40); 464.0 > 216.0 (52)
Spirotetramat‐ketohydroxy	ESI+	7.0	318.0 > 268.0 (20); 318.0 > 214.0 (25)
Spirotetramat‐monohydroxy	ESI+	5.4	304.0 > 254.0 (16); 304.0 > 211.0 (24)
Tebufenozide	ESI+	9.2	353.2 > 297.2 (4); 353.2 > 133.0 (20)

^a^ Electrospray ionization.

^b^ Retention time.

### Quality Assurance and Quality Control

All analyses were performed in a laboratory accredited by the Czech Institute for Accreditation according to ISO/IEC 17025:2017 standard.^33^ The laboratory regularly participates in proficiency testing organized by the European Union Reference Laboratories (EURL).

### Method validation

The analytical method has been validated in accordance with the requirements specified in document SANTE/11312/2021v2.^34^ The validation study was performed by spiking blank apple samples with mixtures of pesticide standards (i.e., MIX A: parent compounds and MIX B: metabolites) at spiking levels of 0.002 mg kg^−1^ and 0.02 mg kg^−1^ in six replicates for each spiking level. After 30 min of pesticide incorporation, the spiked samples were extracted as described in ‘Sample preparation’ section. The performance characteristics obtained from the validation of the analytical method for the targeted analytes were recovery (REC), relative standard deviation for repeatability (RSD_r_), limit of quantification (LOQ), linearity range and matrix effect (ME, see Eqn (1)).

(1) $$\mathrm{ME}=\left(A_{\text{matrix}}/A_{\text{solvent}}-1\right)\times 100\%$$

where ME is matrix effect, *A*_matrix_ is the detector response of the standard in apple extract and *A*_solvent_ is the detector response of the standard in acetonitrile.

### Data analysis

Statistica® software (Language Packs 14.0.0; TIBCO Software Inc., Palo Alto, CA, USA) was used to describe the time trend of pesticide dissipation. The decrease in the residue levels of all pesticides was fitted to a first‐order kinetic model. The kinetic model parameters were calculated using the following equations (see Eqns (2) and (3)):

(2) $$C_{\left(t\right)}=C_{0}\times e^{-\mathrm{kt}}$$

(3) $$t_{1/2}=\left(\text{ln2}\right)/k$$

where *C*_(*t*)_ (in mg kg^−1^) is the pesticide residue concentration at time *t* (in days), *C*_0_ (in mg kg^−1^) is the initial pesticide residue concentration, *k* is the dissipation rate constant (in day^−1^) and *t*_1/2_ is the dissipation half‐life of the pesticide (in days).

The paired sample *t*‐test was used to determine the differences in insecticide dissipation rate among apple varieties. The significance level for the test was set at *α* = 0.05.

## RESULTS AND DISCUSSION

### Results of method validation

The LOQs obtained in the validation study for parent pesticides were in the range of 0.001–0.002 mg kg^−1^. Low LOQs of 0.002 mg kg^−1^ were also achieved in the case of metabolites of spirotetramat. In contrast, metabolites of flonicamid showed a lower quality of detectability compared to other compounds included in this study and thus their LOQs were determined to be in the range of 0.005–0.02 mg kg^−1^. With the exception of spirotetramat‐enol‐glucoside, the calculated RSD_r_ for the remaining analytes was less than 10%. REC of all pesticides tested ranged from 84% to 99%. Differences in response between the standard in apple extract and the standard in solvent expressed as ME were calculated to be in the range of −33% to 42%. Although more than 20% signal suppression or enhancement was observed for only four analytes, matrix‐matched calibration was used throughout the study to compensate for such effects. The results of the method validation are summarized in Table 3, the chromatograms of all analytes at the level corresponding to their LOQ are provided in Fig. S5.

**Table 3:** Performance characteristics for the targeted analytes obtained from the method validation (*n* = 6)

Pesticide/metabolite	LOQ^a^ (mg kg^−1^)	0.002 mg kg^−1^		0.02 mg kg^−1^		ME^d^ (%)	Linearity (mg kg^−1^)
		REC^b^ (%)	RSD_r_ ^c^ (%)	REC (%)	RSD_r_ (%)		
Acetamiprid	0.001	88	8	89	6	−13	0.001–0.05
Chlorantraniliprole	0.002	89	9	93	5	13	0.002–0.05
Cyantraniliprole	0.001	92	4	92	4	4	0.001–0.05
Flonicamid	0.002	89	6	90	5	−29	0.002–0.05
Flonicamid metabolite: TFNA	0.02	—	—	89	8	1	0.02–0.1
Flonicamid metabolite: TFNG	0.005	—	—	95	10	−1	0.005–0.1
Flupyradifurone	0.001	90	5	94	5	23	0.001–0.05
Pirimicarb	0.001	84	5	84	4	−33	0.001–0.05
Pyriproxyfen	0.001	86	8	85	6	−19	0.001–0.05
Spinosyn A	0.002	95	6	87	6	4	0.002–0.05
Spinosyn D	0.002	87	10	89	7	−3	0.002–0.05
Spirotetramat	0.001	93	7	92	6	17	0.001–0.05
Spirotetramat‐enol	0.002	84	10	88	5	9	0.002–0.05
Spirotetramat‐enol glucoside	0.002	90	11	89	9	−17	0.002–0.05
Spirotetramat‐ketohydroxy	0.002	85	9	91	7	10	0.002–0.05
Spirotetramat‐monohydroxy	0.002	98	10	99	8	42	0.002–0.05
Tebufenozide	0.001	87	9	83	9	4	0.001–0.05

^a^ Limit of quantification.

^b^ Recovery.

^c^ Relative standard deviation for repeatability.

^d^ Matrix effect.

### Dissipation of insecticides in the apple orchard

The dissipation study included ten major insecticides used to control major apple pests (see Table S1). Apple trees of two varieties (Rosana and Selena) in the experimental orchard were treated with insecticidal preparations during the monitoring studies conducted from 2020 to 2023. Dissipation of each active ingredient in apple samples was monitored over time for 1–55 days after PPP application in 2020–2021 and for 1–101 days after PPP application in 2022–2023, depending on the date of pesticide treatment. Due to a significant variation in residue concentrations, mathematical models with significant values of coefficient of determination (*R* ^2^ > 0.5) could not be developed for acetamiprid, pirimicarb and pyriproxyfen in at least 1 year of field trials (treatments in the period from the end of July to mid‐August). Lower *R* ^2^ values were also obtained for later applied flonicamid and spirotetramat, for which the sum with their metabolites was calculated. The dissipation of spirotetramat, flonicamid and their selected metabolites is discussed in more detail in the following sections. Coefficients of determination for the other insecticides tested were in the range of 0.51 and 0.99 (median of *R* ^2^: 0.96 (Rosana) and 0.98 (Selena)). The measured and simulated residual concentrations determined for all tested insecticides in apples are shown in Figs S6–S15. The kinetic parameters of the mathematical models and the calculated dissipation half‐lives for all insecticides are summarized in Table 4.

**Table 4:** Kinetic model parameters (*C*_0_, *k*), dissipation half‐lives (*t*_1/2_), calculated residue concentrations at the end of the preharvest interval (PHI) (*C*_(PHI)_) and determined residue concentrations at harvest (*C*_(harvest)_)

Active ingredient	PHI (day)	MRL^a^ (mg kg^−1^)	Year	Apple variety ‘Rosana’						Apple variety ‘Selena’					
				Kinetic model parameters						Kinetic model parameters					
				*C*_0_ (mg kg^−1^)	*k* (day^−1^)	*R* ^2^ ^b^	*t*_1/2_ (day)	*C*_(PHI)_ (mg kg^−1^)	*C*_(harvest)_ (mg kg^−1^)	*C*_0_ (mg kg^−1^)	*k* (day^−1^)	*R* ^2^	*t*_1/2_ (day)	*C*_(PHI)_ (mg kg^−1^)	*C*_(harvest)_ (mg kg^−1^)
Acetamiprid	14	0.4	2020	0.0859	0.0308	0.82	22.5	0.056	0.022	0.1060	0.0354	0.91	19.6	0.065	0.028
			2021	nsm^f^	—	0.03	—	—	0.053	nsm	—	0.49	—	—	0.055
			2022	0.0924	0.0226	0.93	30.7	0.067	0.017	0.0935	0.0200	0.98	34.7	0.071	0.015
			2023	0.1216	0.0377	0.96	18.4	0.072	0.015	0.1183	0.0277	0.98	25.1	0.080	0.020
Cyantraniliprole	7	0.8	2021	0.0728	0.0604	0.87	11.5	0.048	0.004	0.1139	0.0919	0.99	7.5	0.060	0.002
			2022	0.1051	0.1165	0.98	6.0	0.047	0.002	0.1134	0.1122	0.98	6.2	0.052	0.003
			2023	0.1175	0.1278	0.99	5.4	0.048	0.001	0.1122	0.1619	0.99	4.3	0.036	0.001
Chlorantraniliprole	14	0.4	2020	0.0500	0.0316	0.77	22.0	0.032	0.016	0.0649	0.0294	0.96	23.6	0.043	0.020
			2021	0.0601	0.0504	0.93	13.7	0.030	0.011	0.0609	0.0357	0.59	19.4	0.037	0.027
			2022	0.0701	0.0611	0.99	11.3	0.030	0.003	0.0907	0.0692	0.99	10.0	0.034	0.003
			2023	0.0807	0.0397	0.99	17.4	0.046	0.008	0.0751	0.0341	0.99	20.3	0.047	0.008
Flonicamid (sum)^**c**^	21	0.3	2020	nsm	**—**	0.36	—	—	0.094	nsm	—	0.01	—	—	0.199
			2022	0.1309	0.0129	0.65	53.6	0.100	0.017	0.1087	0.0115	0.59	60.4	0.085	0.019
			2023	0.1219	0.0088	0.90	79.1	0.101	0.061	0.1448	0.0105	0.74	66.3	0.116	0.061
Flonicamid (parent)	—	—	2020	0.1559	0.0483	0.82	14.3	—	0.053	0.1402	0.0195	0.71	35.5	—	0.076
			2022	0.1352	0.0327	0.94	21.2	—	0.017	0.1053	0.0216	0.93	32.2	—	0.019
			2023	0.1245	0.0358	0.98	19.4	—	0.015	0.1279	0.0272	0.98	25.5	—	0.015
Flupyradifurone	14	0.6	2021	0.1487	0.0227	0.80	30.6	0.108	0.060	0.1634	0.0136	0.90	51.1	0.135	0.079
			2022	0.1836	0.0546	0.98	12.7	0.086	0.010	0.2341	0.0307	0.95	22.6	0.152	0.030
			2023	0.3328	0.1219	0.99	5.7	0.060	0.003	0.3194	0.0515	0.99	13.5	0.155	0.011
Pirimicarb	7	0.5	2020	0.1749	0.0327	0.87	21.2	0.139	0.047	0.1875	0.0297	0.73	23.3	0.152	0.065
			2021	0.1063	0.0415	0.87	16.7	0.080	0.034	nsm	**—**	0.09	—	—	0.052
			2022	0.2602	0.0335	0.96	20.7	0.206	0.020	0.3575	0.0315	0.99	22.0	0.287	0.019
			2023	0.4006	0.0406	0.98	17.1	0.301	0.029	0.3242	0.0289	0.99	24.0	0.265	0.035
Pyriproxyfen	−/98	0.2/0.05	2020	nsm	**—**	0.43	—	—	0.129	0.2741	0.0322	0.65	21.5	0.012	0.083
			2021	0.0903	0.0210	0.51	33.0	0.012	0.054	nsm	**—**	0.02	—	—	0.086
			2022	0.1107	0.0430	0.97	16.1	0.002	0.007	0.1084	0.0360	0.97	19.3	0.003	0.010
			2023	0.2518	0.0589	0.99	11.8	0.001	0.010	0.2188	0.0468	0.99	14.8	0.002	0.006
Spinosad^d^	7	0.3	2020	0.0193	0.1115	0.95	6.2	0.009	0.003	0.0332	0.1540	0.97	4.5	0.011	0.003
			2021	0.0443	0.1659	0.98	4.2	0.014	0.003	0.0380	0.1543	0.98	4.5	0.013	0.003
Spirotetramat (sum)^**e**^	21	0.7	2020	0.5137	0.0900	0.97	7.7	0.078	0.035	0.4048	0.0435	0.99	15.9	0.162	0.068
			2021	nsm	—	0.34	—	—	0.079	0.2111	0.0214	0.65	32.4	0.135	0.072
			2022	0.4865	0.0872	0.99	8.0	0.078	0.003	0.5228	0.0644	0.98	10.8	0.135	0.010
			2023	0.3774	0.0729	0.99	9.5	0.082	0.004	0.3073	0.0494	0.99	14.0	0.109	0.013
Spirotetramat (parent)	—	—	2020	0.5421	0.1918	0.99	3.6	—	0.011	0.4260	0.1598	0.99	4.3	—	0.005
			2021	0.1196	0.0506	0.98	13.7	—	0.006	0.1908	0.0688	0.95	10.1	—	0.010
			2022	0.6202	0.2269	0.99	3.1	—	0.001	0.6529	0.2165	0.99	3.2	—	0.001
			2023	0.3722	0.1216	0.99	5.7	—	0.001	0.3044	0.1035	0.99	6.7	—	0.003
Tebufenozide	32	1.0	2022	0.2160	0.0266	0.95	26.1	0.092	0.027	0.2432	0.0250	0.91	27.7	0.109	0.040
			2023	0.3283	0.0656	0.99	10.6	0.040	0.013	0.3003	0.0544	0.98	12.7	0.053	0.015

^a^ Maximum residue levels apply to apples (product code 0130010).^35^

^b^ Significant model (*R* ^2^ > 0.5).

^c^ Flonicamid (sum of flonicamid, TFNA and TFNG expressed as flonicamid).

^d^ Spinosad (spinosad, sum of spinosyn A and spinosyn D).

^e^ Spirotetramat and spirotetramat‐enol (sum of), expressed as spirotetramat.

^f^ non‐significant model.

The dissipation half‐lives in apples ranged from 3 to 5 days (cyantraniliprole in 2023, spinosad in 2021 and spirotetramat (parent) in 2020 and 2022) to 66–79 days (sum of flonicamid and its metabolites in 2023). Calculated mean dissipation half‐lives were determined with standard deviations of 1–9 days for most of the insecticides tested during the 4‐year monitoring study. Standard deviations of the *t*_1/2_ > 10 days were calculated for flupyradifurone, flonicamid, pyriproxyfen and tebufenozide.

The developed mathematical models were used to predict the concentration of residues at the end of the PHI of each pesticide preparation. PHIs are mandatory for each pesticide product, but are not (and cannot be) uniform worldwide. In the Czech Republic, PHIs are set by the Central Institute for Supervising and Testing in Agriculture.^29^ The calculated concentrations of pesticide residues in apples were compared with the MRLs established in the consolidated version of Regulation (EC) No 396/2005.^35^ The concentrations of insecticides were at levels corresponding to 2–26% of the MRLs in both apple varieties with the exception of flonicamid (sum) and pirimicarb, whose concentrations were calculated at levels corresponding to 28–39% and up to 60% of the established MRLs, respectively (see Table 4). The calculated concentrations of pesticide residues at the end of the PHI were comparable in both pesticide treatment regimes (i.e., early and late pesticide application) which is probably caused by the lack of extremes in temperature or precipitation among the experimental years. As pesticide applications in 2022 and 2023 were carried out from mid‐June to the end of June, the time for pesticide dissipation before harvest was about twice as long as in 2020 and 2021, when the treatments were carried out from the end of July to mid‐August. Residue levels found in apples at harvest were significantly lower in the 2022 and 2023 experimental years than in 2020 and 2021, moreover, in several cases the residue concentrations were quantified even below the 0.01 mg kg^−1^ limit (see Table 4).

In some years, the PHI for several PPPs and MRLs was also changed while conducting the field trial. In the case of pyriproxyfen, the MRL applicable to apples was lowered from 0.2 to 0.05 mg kg^−1^ in 2023. In addition, the PHI for pyriproxyfen‐based pesticide preparation was not specified at the beginning of this study and was later set at 98 days. Table 4 also shows the concentration of residues found in apple samples collected at harvest time. Residues of all insecticides tested were found at levels below current MRLs in both pesticide treatment regimes.

Our results are in agreement with other published studies focused on pesticide residues in apples under field conditions for the following compounds: acetamiprid, chlorantraniliprole and pirimicarb. For instance, in the case of acetamiprid, the mean values of dissipation half‐lives obtained in the field trials (Rosana: 23.8 ± 6.3 days, Selena: 26.5 ± 7.6 days) are comparable to those obtained in the study by Alister *et al*., where the *t*_1/2_ was determined to be 22.4 ± 7.6 days considering the apple diameter > 40.5 mm.^25^ In Poland, two studies aimed to monitor the dissipation of chlorantraniliprole in apples. Half concentration of the residues was experimentally measured 16–17 days after treatment^26^ and 14 days after treatment,^27^ respectively. These results correspond to those obtained in our study (Rosana: 16.1 ± 4.6 days, Selena: 18.3 ± 5.8 days). Studies on the dissipation of pirimicarb in apples by Podbielska *et al*.^5^ showed that the half‐life was reached 16.6 days after treatment. In our study with pirimicarb, comparable *t*_1/2_ values were obtained, that is, 18.9 ± 2.4 days (Rosana) and 23.1 ± 1.0 days (Selena). It should be noted that slight differences in the rate of pesticide dissipation could be influenced not only by the climatic and weather conditions in the orchard, the application technique of the pesticide preparations or the phenological phase in which the treatment was carried out, but also by the morphological characteristics of the respective apple variety.

However, no studies on the dissipation kinetics of cyantraniliprole, flupyradifurone, pyriproxyfen, spinosad, and tebufenozide in apples under field conditions have been available for comparison with our data. From the experimental years 2022 and 2023 (pesticide application in June), the calculated *t*_1/2_ values for pyriproxyfen were 11.8–16.1 days (Rosana) and 14.8–19.3 days (Selena). The dissipation of this insecticide was also studied in our previous study where pears were treated under comparable conditions. The mean values of dissipation half‐lives obtained in the field trials on pears (9.7–11.3 days) were slightly lower than those obtained for apples.^32^ Based on our results, cyantraniliprole (Rosana: *t*_1/2_ = 7.6 ± 3.4 days, Selena: *t*_1/2_ = 6.0 ± 1.6 days) and spinosad (Rosana: *t*_1/2_ = 5.2 ± 1.4 days, Selena: *t*_1/2_ = 4.5 ± 0.0 days) were among the insecticides with the shortest dissipation half‐lives from all application regimes (see Fig. 2). In tomatoes, the study by Malhat *et al*.^36^ calculated a dissipation half‐life of 2.6 days for cyantraniliprole and a dissipation half‐life of 3.74 to 4.71 days for spinosad (depending on the dose applied) in the study by Adak and Mukherjee.^37^ These results confirm the fast dissipation of spinosad independently of the crop under field conditions, mainly due to photodegradation.^37^ There are no studies focusing on the dissipation of flupyradifurone in fruits. To date, only three studies have been published on ginseng,^38^ mustard^39^ and pepper.^40^

### Differences in pesticide dissipation between apple varieties Rosana and Selena

In this study, the differences in pesticide dissipation rate between two apple varieties (Rosana and Selena) were investigated. Rosana and Selena are late fall/early winter apple varieties. Mature apples are typically harvested from mid‐September (Rosana) to the end of September (Selena).^41^

**Figure 3:**
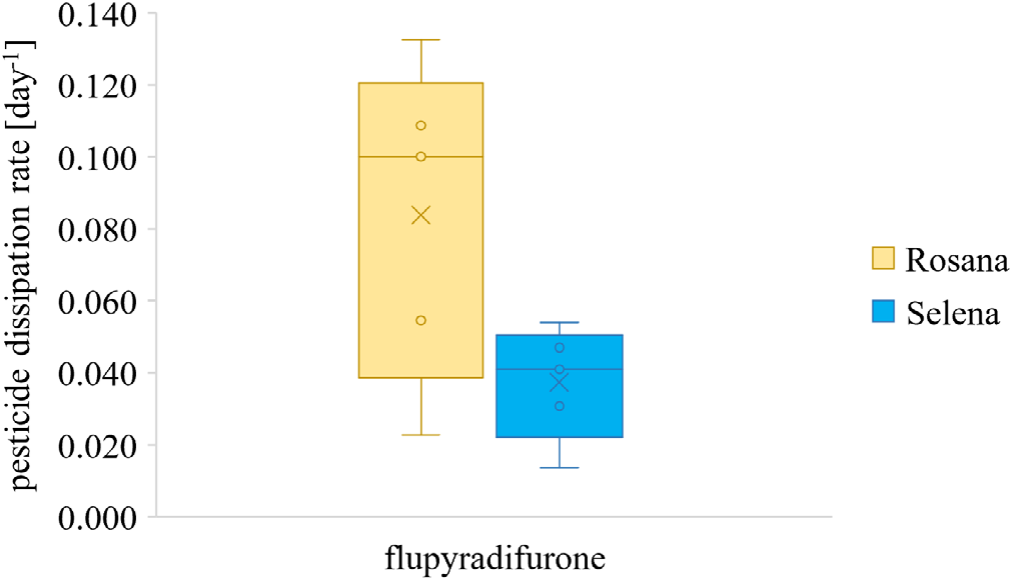
Calculated dissipation rates of flupyradifurone in apple varieties Rosana and Selena.

In the case of flupyradifurone, a statistically significant difference in dissipation rates between the tested apple varieties Rosana and Selena was found using the paired sample *t*‐test (*P*‐value 0.023). The dissipation rates calculated from the measured data were 1.7–2.5 times lower in Selena than in Rosana (see Fig. 3). The difference in dissipation rate can be caused by the difference in morphology of both apple varieties. Apple variety Rosana has a skin with a slightly thinner epicuticular wax layer compared to apple variety Selena, which may affect the extent of flupyradifurone penetration into the apple and thus its degradation. Apple varieties also differ in their genome which is closely related to the ability to resist biotransformation in plants. No statistically significant difference in dissipation rates was found for the other insecticides tested in this study.

### The fate of spirotetramat in apples under field conditions

In the Czech Republic, PPPs containing spirotetramat are registered in apples for the control of insects belonging to the family *Aphididae* (e.g., green apple aphid or rosy apple aphid), the family *Pemphigidae* (e.g., woolly apple aphid), and the family *Diaspilidae* (e.g., oystershell scale (*Lepidosaphes ulmi*) or San Jose scale (*Quadraspidiotus perniciosus*)).

**Figure 4:**
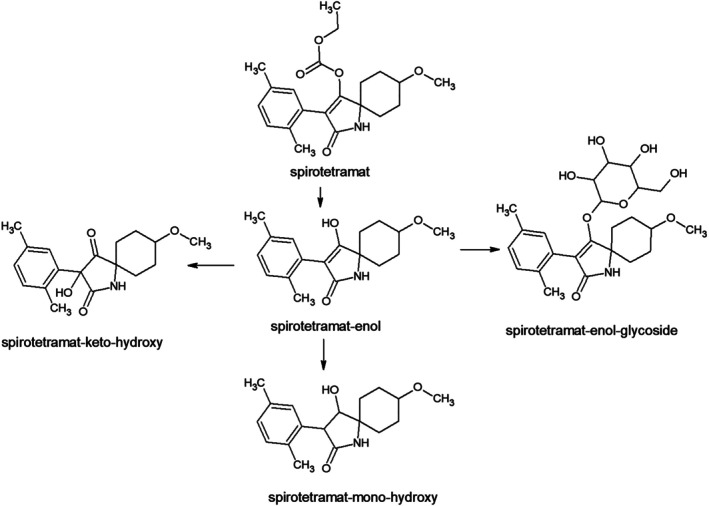
Metabolic pathways of spirotetramat in plants.

In this study, spirotetramat and its four metabolites, spirotetramat‐enol, spirotetramat‐enol glucoside, spirotetramat‐ketohydroxy and spirotetramat‐monohydroxy, were monitored. The metabolite spirotetramat‐enol is formed by hydrolysis of spirotetramat in the first phase of biotransformation in plants. This compound is an intermediate in reactions (oxidation–reduction reaction, glycosylation) leading to the formation of the other three screened metabolites, see Fig. 4.

**Figure 5:**
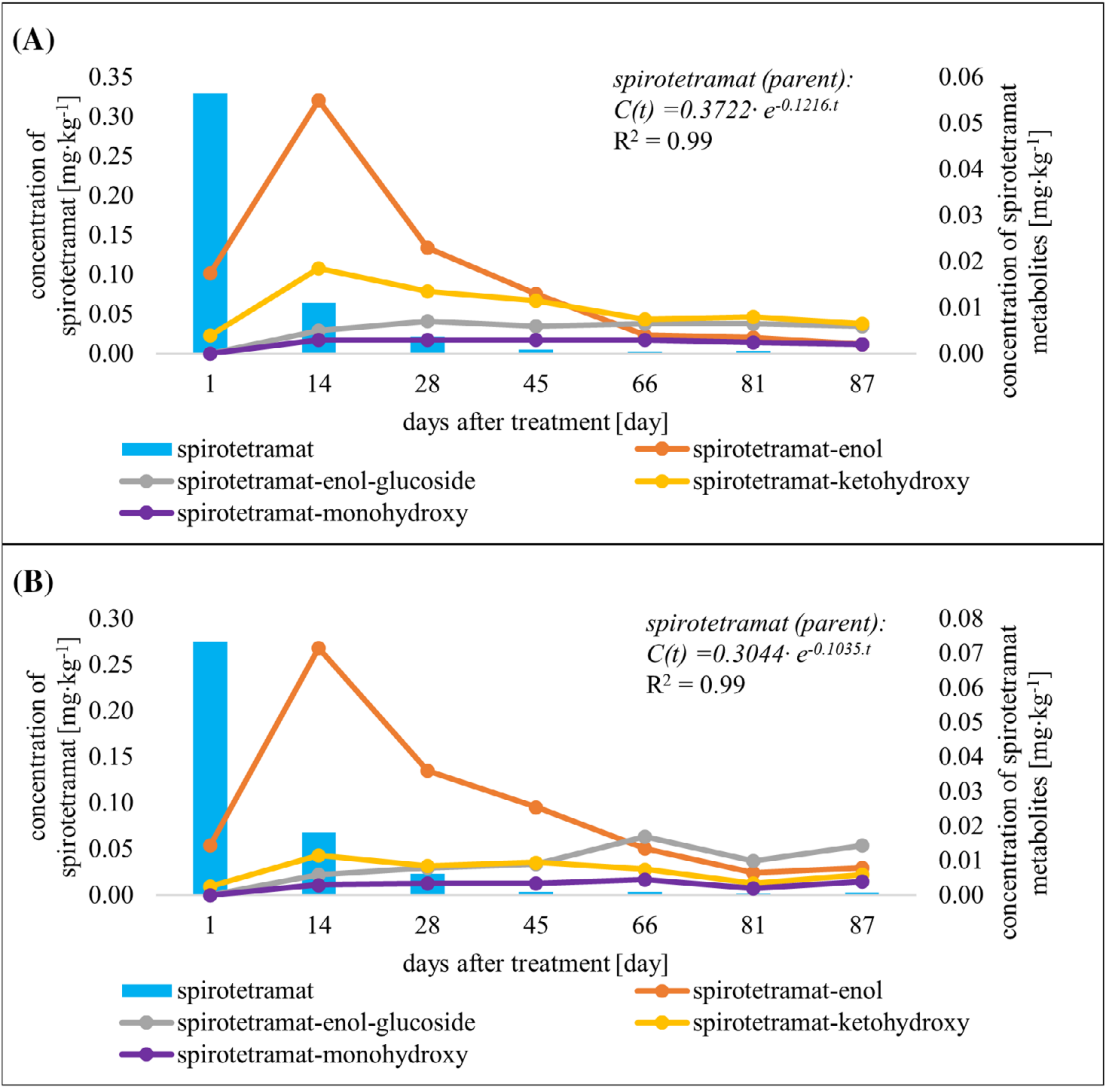
Dissipation curves of spirotetramat and its metabolites in apple varieties Rosana (A) and Selena (B) in 2023.

Based on the results, a relatively high dissipation rate of spirotetramat was observed (mean value of *t*_1/2_ was 6.3 ± 3.8 days). The fate of spirotetramat was similar in both apple varieties tested. While on day 1 after treatment, a major portion of the residues were detected as the parent insecticide (88–90% of the initial residue concentration) and only low concentrations of two metabolites, spirotetramat‐enol (0.015–0.018 mg kg^−1^) and spirotetramat‐ketohydroxy (0.003–0.004 mg kg^−1^), were found, on day 3, a significant increase in the concentrations of spirotetramat‐enol was observed, the levels of this metabolite were determined in the range of 0.040–0.079 mg kg^−1^. On days 14–18 after treatment, the maximum concentrations of spirotetramat‐enol were found in apple samples (0.055–0.132 mg kg^−1^). Although the fate of spirotetramat‐ketohydroxy in apples was similar to that of spirotetramat‐enol, detected concentrations were much lower. However, the metabolites spirotetramat‐enol glucoside and spirotetramat‐monohydroxy were detectable from day 14 after treatment and their maximum concentrations in apples were found in samples collected from 44 to 66 days after treatment. The dissipation curves of spirotetramat and its metabolites obtained in 2023 from both apple varieties are shown in Fig. 5. The results of residue analysis of these compounds in apples from other experimental years are shown in Figs S16–S18.

By 2021, the other three metabolites have been included in the residue definition,^35^ however, spirotetramat and its metabolite spirotetramat‐enol have been found to be sufficient markers in all crops representing the majority of residues.^42^ According to the consolidated version of Regulation (EC) No 396/2005, the residue definition of spirotetramat is defined as ‘spirotetramat and spirotetramat‐enol (sum of), expressed as spirotetramat’.^35^ Taking into account this definition, the calculated mean dissipation half‐life of spirotetramat (sum) has been extended from 6.3 days to 14.0 days.

The fate of spirotetramat in apples was published in the study of Salazar‐López *et al*. The four metabolites of spirotetramat mentioned earlier were detected in apples treated with this insecticide. However, a direct comparison of the detected concentrations or their ratios is not possible due to the differences in pesticide treatment (differences in application doses and number of applications).^43^ However, the dissipation of this insecticide was also studied in our previous study aimed at pears. Based on the results, slightly higher dissipation half‐life values of spirotetramat (sum) were observed in apples compared to pears (pears: *t*_1/2_ = 3.4–9.2 days).^32^ Although the fate of the pesticide may differ among the different plant species, both apples and pears are included in the pome fruits and dissipation is expected to be comparable. In China, changes in the concentrations of spirotetramat with its four metabolites were investigated in field trials on peaches, where the dissipation half‐life values of spirotetramat (parent) *t*_1/2_ = 6.24–7.07 days were calculated. Comparable results in terms of concentration ratios and dissipation patterns were observed for its metabolites.^44^

### The fate of flonicamid in apples under field conditions

**Figure 6:**
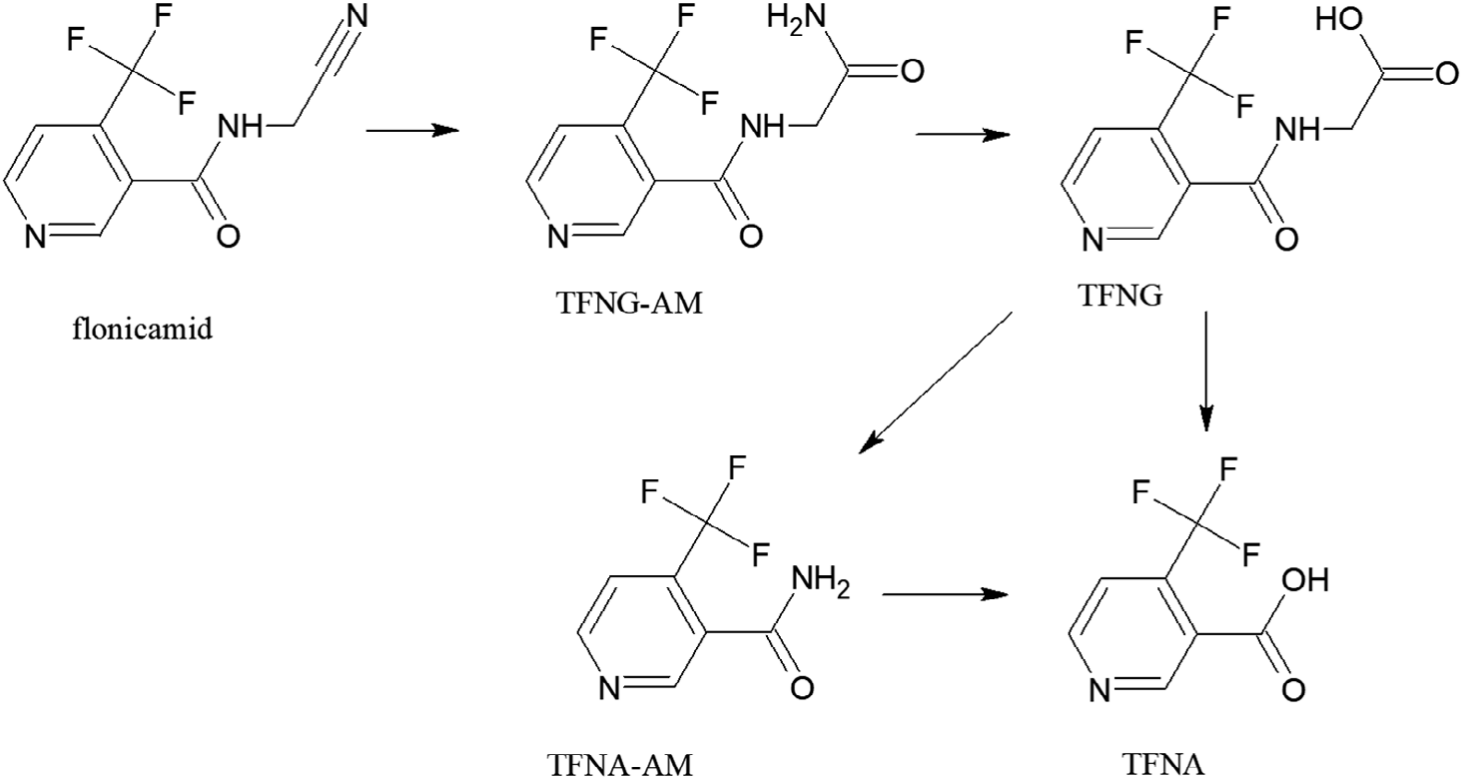
Metabolic pathways of flonicamid in plants.

In the Czech Republic, PPPs containing flonicamid are registered for the control of insects belonging to the family *Aphididae* in apples. Although four major metabolites of flonicamid (TFNG, TFNG‐AM, TFNA and TFNA‐AM) are known, only two of them were monitored in this study. The metabolic pathway of flonicamid in plants is shown in Fig. 6.^45^ The monitored metabolites TFNA and TFNG are included in the residue definition according to Regulation (EC) No 396/2005: ‘flonicamid (sum of flonicamid, TFNA and TFNG expressed as flonicamid)’.^35^

**Figure 7:**
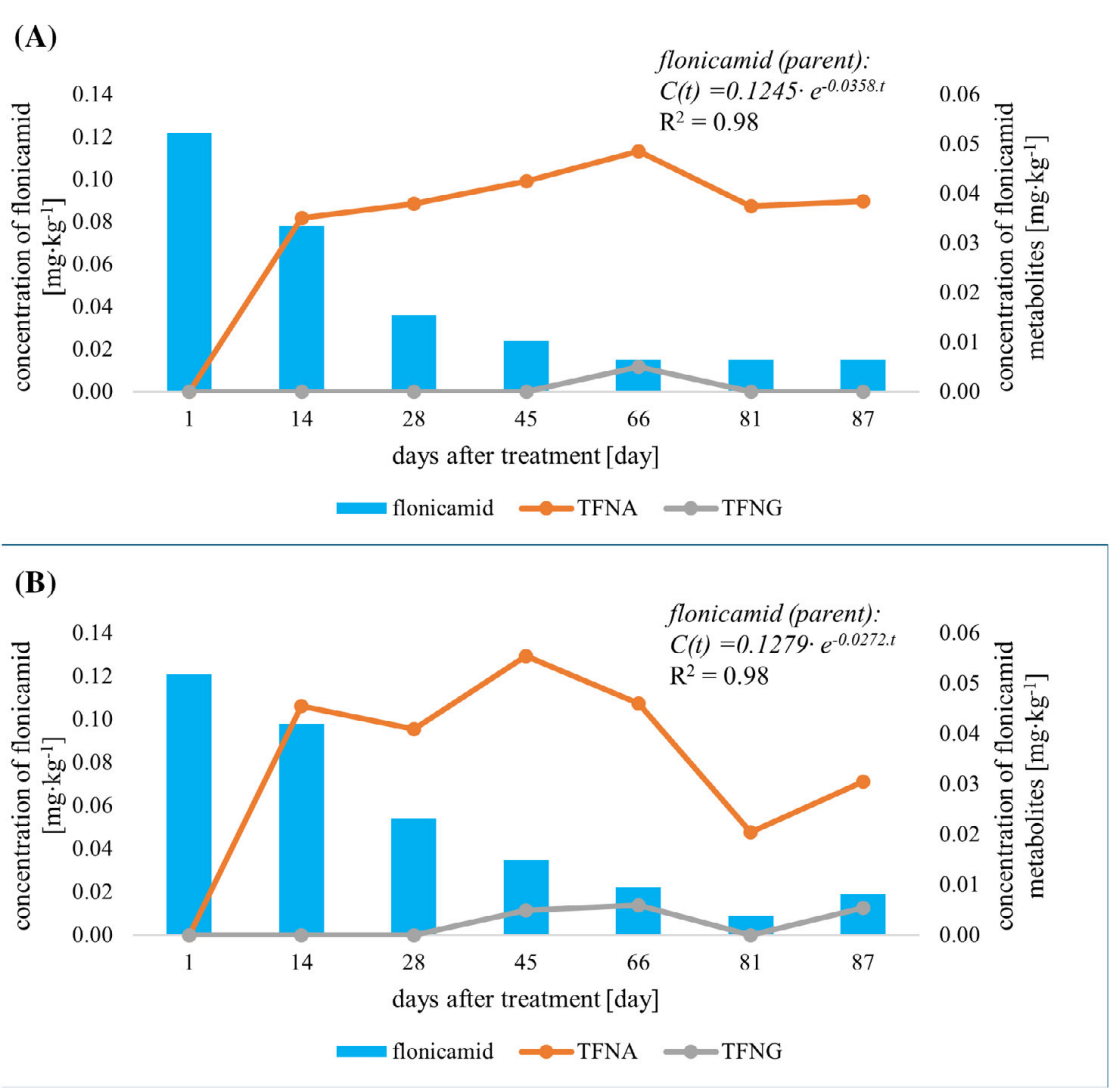
Dissipation curves of flonicamid and its metabolites in apple varieties Rosana (A) and Selena (B) in 2023.

Based on the results of the 4‐year field trials, the mean dissipation half‐life of flonicamid (parent compound) in apples was calculated to be *t*_1/2_ = 18.3–31.0 days. The metabolite TFNG was detected only in several apple samples at concentrations not exceeding a level of 0.01 mg kg^−1^. The metabolite TFNA was detected at quantifiable levels (> 0.02 mg kg^−1^) from day 14 after treatment with flonicamid. Concentrations of this metabolite were in the range of 0.020–0.056 mg kg^−1^ in apple samples. The fate of flonicamid was comparable in both tested apple varieties, the dissipation curves of parent insecticide and its metabolites obtained in 2023 are shown in Fig. 7. Considering the residue definition, the calculated mean dissipation half‐life of flonicamid (sum) has been fairly extended from 18.3–31.0 days to 63.3–66.4 days. The results of residue analysis of these compounds in apples from other experimental years are shown in Figs S19 and S20.

The fate of flonicamid in apples was investigated in the study by Liu *et al*., where the dissipation half‐lives were determined to be approximately 3–4 times lower (*t*_1/2_ = 5.1–6.1 days) than in our study. The higher dissipation rate was probably observed due to the different climatic zone and higher temperatures during the experimental period.^46^

## CONCLUSIONS

The dissipation of ten currently registered insecticides in two apple varieties (Rosana and Selena) was described in a monitoring study over a period of 4 years using a first‐order kinetic equation. The data collected during the 4 years of the experiment cover different conditions in the apple orchard (e.g., weather conditions) and thus variability in insecticide dissipation and its influence on residue concentration in apples. The mean calculated dissipation half‐lives ranged from 4.5 to 66.4 days. In both apple varieties, spinosad showed the highest dissipation rate in apples while flonicamid showed the lowest rate. With the exception of flupyradifurone, the dissipation rates of the other insecticides were not found to be significantly different between the apple varieties tested.

In the case of flonicamid and spirotetramat, the fate of the parent pesticides was monitored together with their selected metabolites. As the metabolites TFNG, TFNA and spirotetramat‐enol are included in the residue definition according to Regulation (EC) No 396/2005, the time needed to reduce the levels to an expected concentration is 2–3 times longer than for the parent pesticides.

From 2023, the levels of pesticide residues in pome fruits grown in the IPM practice in the Czech Republic must comply with the requirements of Government Regulation No. 80/2023 Coll., which sets a level corresponding to 30% of the MRL from Regulation (EC) No. 396/2005 as the maximum tolerable concentration of residues in/on fruit. For eight out of ten insecticides (except flonicamid (sum) and pirimicarb), residue concentrations at the end of the PHI for each pesticide preparation were below the action threshold of 30% of the MRL. The results of this study provide a scientific basis for the appropriate selection and use of pesticides by orchardists growing apples under the IPM.

## AUTHOR CONTRIBUTIONS

Dana Schusterova: investigation, formal analysis, writing – original draft, writing – review and editing; Jitka Stara: conceptualization, methodology; Frantisek Kocourek: resources, methodology; Vojtech Hrbek: formal analysis, writing – review and editing; Petr Mraz: formal analysis, validation; Vit Kosek: formal analysis, visualization; Petra Vackova: formal analysis, validation; Vladimir Kocourek: resources, writing – review and editing; Jana Hajslova: funding acquisition, conceptualization, methodology; Tereza Horska: investigation, writing – original draft.

## CONFLICT OF INTEREST STATEMENT

Declarations of interest: none.

## Supporting information


**Figure S1.** Weather conditions in the apple orchard during the field trials in 2020.
**Figure S2.** Weather conditions in the apple orchard during the field trials in 2021.
**Figure S3.** Weather conditions in the apple orchard during the field trials in 2022.
**Figure S4.** Weather conditions in the apple orchard during the field trials in 2023.
**Figure S5.** LC–MS chromatograms of pesticides and their selected metabolites at levels corresponding to their LOQ.
**Figure S6.** Pesticide dissipation curves of acetamiprid in apples from 2020 (A) and 2021 (B) – later pesticide applications; from 2022 (C) and 2023 (D) – earlier pesticide applications.
**Figure S7.** Pesticide dissipation curves of chlorantraniliprole in apples from 2020 (A) and 2021 (B) – later pesticide applications; from 2022 (C) and 2023 (D) – earlier pesticide applications.
**Figure S8.** Pesticide dissipation curves of cyantraniliprole in apples and 2021 (A) – later pesticide applications; from 2022 (B) and 2023 (C) – earlier pesticide applications.
**Figure S9.** Pesticide dissipation curves of flonicamid (parent) in apples and 2020 (A) – later pesticide applications; from 2022 (B) and 2023 (C) – earlier pesticide applications.
**Figure S10.** Pesticide dissipation curves of flupyradifurone in apples and 2021 (A) – later pesticide applications; from 2022 (B) and 2023 (C) – earlier pesticide applications.
**Figure S11.** Pesticide dissipation curves of pirimicarb in apples from 2020 (A) and 2021 (B) – later pesticide applications; from 2022 (C) and 2023 (D) – earlier pesticide applications.
**Figure S12.** Pesticide dissipation curves of pyriproxyfen in apples from 2020 (A) and 2021 (B) – later pesticide applications; from 2022 (C) and 2023 (D) – earlier pesticide applications.
**Figure S13.** Pesticide dissipation curves of spinosad in apples from 2020 (A) and 2021 (B) – later pesticide applications.
**Figure S14.** Pesticide dissipation curves of tebufenozide in apples from 2022 (A) and 2023 (B) – earlier pesticide applications.
**Figure S15.** Pesticide dissipation curves of spirotetramat (parent) in apples from 2020 (A) and 2021 (B) – later pesticide applications; from 2022 (C) and 2023 (D) – earlier pesticide applications.
**Figure S16.** Dissipation curves of spirotetramat and its metabolites in apple varieties Rosana (A) and Selena (B) in 2020.
**Figure S17.** Dissipation curves of spirotetramat and its metabolites in apple varieties Rosana (A) and Selena (B) in 2021.
**Figure S18.** Dissipation curves of spirotetramat and its metabolites in apple varieties Rosana (A) and Selena (B) in 2022.
**Figure S19.** Dissipation curves of flonicamid and its metabolites in apple varieties Rosana (A) and Selena (B) in 2020.
**Figure S20.** Dissipation curves of flonicamid and its metabolites in apple varieties Rosana (A) and Selena (B) in 2022.
**Table S1.** The range of authorized uses of tested plant protection products for apple trees in the Czech Republic.
**Table S2.** The detailed timetable of pesticide treatments in a 4‐year field trial.

## Data Availability

The data that support the findings of this study are available from the corresponding author upon reasonable request.
