# Supplementary material for: Dissipation study of ten insecticides in apples under field conditions
Source: J Sci Food Agric. 2025 May 12;105(12):6603–14. doi: 10.1002/jsfa.14370 (PMC12355340; doi:10.1002/jsfa.14370)
Supplement: Supplementary file 1 — Figure S1. Weather conditions in the apple orchard during the field trials in 2020. Figure S2. Weather conditions in the apple orchard during the field trials in 2021. Figure S3. Weather conditions in the apple orchard during the field trials in 2022. Figure S4. Weather conditions in the apple orchard during the field trials in 2023. Figure S5. LC–MS chromatograms of pesticides and their selected metabolites at levels corresponding to their LOQ. Figure S6. Pesticide dissipation curves of acetamiprid in apples from 2020 (A) and 2021 (B) – later pesticide applications; from 2022 (C) and 2023 (D) – earlier pesticide applications. Figure S7. Pesticide dissipation curves of chlorantraniliprole in apples from 2020 (A) and 2021 (B) – later pesticide applications; from 2022 (C) and 2023 (D) – earlier pesticide applications. Figure S8. Pesticide dissipation curves of cyantraniliprole in apples and 2021 (A) – later pesticide applications; from 2022 (B) and 2023 (C) – earlier pesticide applications. Figure S9. Pesticide dissipation curves of flonicamid (parent) in apples and 2020 (A) – later pesticide applications; from 2022 (B) and 2023 (C) – earlier pesticide applications. Figure S10. Pesticide dissipation curves of flupyradifurone in apples and 2021 (A) – later pesticide applications; from 2022 (B) and 2023 (C) – earlier pesticide applications. Figure S11. Pesticide dissipation curves of pirimicarb in apples from 2020 (A) and 2021 (B) – later pesticide applications; from 2022 (C) and 2023 (D) – earlier pesticide applications. Figure S12. Pesticide dissipation curves of pyriproxyfen in apples from 2020 (A) and 2021 (B) – later pesticide applications; from 2022 (C) and 2023 (D) – earlier pesticide applications. Figure S13. Pesticide dissipation curves of spinosad in apples from 2020 (A) and 2021 (B) – later pesticide applications. Figure S14. Pesticide dissipation curves of tebufenozide in apples from 2022 (A) and 2023 (B) – earlier pesticide applications. Figure S15. Pestici [file JSFA-105-6603-s001.pdf]

Supporting Information for:

Dissipation study of 10 insecticides in apples under field conditions

Dana Schusterova[1]; Jitka Stara[2]; Frantisek Kocourek[2]; Vojtech Hrbek[1]; Petr Mraz[1]; Vit Kosek[1]; Petra Vackova[1], Vladimir Kocourek[1]; Jana Hajslova[1]; Tereza Horska[2]

[1] University of Chemistry and Technology, Prague, Faculty of Food and Biochemical Technology, Department of Food Analysis and Nutrition, Technicka 3, 166 28 Prague 6, Czech Republic

[2] Czech Agrifood Research Center, Drnovska 507/73, 161 06 Prague 6, Czech Republic

Tereza Horska (**corresponding author**): tereza.horska@carc.cz; https://orcid.org/0000-0001-7557-1586, Czech Agrifood Research Center, Drnovska 507/73, 161 06 Prague 6, Czech Republic

FIGURE CAPTIONS:

Figure S1. Weather conditions in the apple orchard during the field trials in 2020.

Figure S2. Weather conditions in the apple orchard during the field trials in 2021.

Figure S3. Weather conditions in the apple orchard during the field trials in 2022.

Figure S4. Weather conditions in the apple orchard during the field trials in 2023.

Figure S5. LC-MS chromatograms of pesticides and their selected metabolites at levels corresponding to their LOQ.

Figure S6. Pesticide dissipation curves of acetamiprid in apples from 2020 (A) and 2021 (B) – later pesticide applications; from 2022 (C) and 2023 (D) – earlier pesticide applications.

Figure S7. Pesticide dissipation curves of chlorantraniliprole in apples from 2020 (A) and 2021 (B) – later pesticide applications; from 2022 (C) and 2023 (D) – earlier pesticide applications.

Figure S8. Pesticide dissipation curves of cyantraniliprole in apples and 2021 (A) – later pesticide applications; from 2022 (B) and 2023 (C) – earlier pesticide applications.

Figure S9. Pesticide dissipation curves of flonicamid (parent) in apples and 2020 (A) – later pesticide applications; from 2022 (B) and 2023 (C) – earlier pesticide applications.

Figure S10. Pesticide dissipation curves of flupyradifurone in apples and 2021 (A) – later pesticide applications; from 2022 (B) and 2023 (C) – earlier pesticide applications.

Figure S11. Pesticide dissipation curves of pirimicarb in apples from 2020 (A) and 2021 (B) – later pesticide applications; from 2022 (C) and 2023 (D) – earlier pesticide applications.

Figure S12. Pesticide dissipation curves of pyriproxyfen in apples from 2020 (A) and 2021 (B) – later pesticide applications; from 2022 (C) and 2023 (D) – earlier pesticide applications.

Figure S13. Pesticide dissipation curves of spinosad in apples from 2020 (A) and 2021 (B) – later pesticide applications.

Figure S14. Pesticide dissipation curves of tebufenozide in apples from 2022 (A) and 2023 (B) – earlier pesticide applications.

Figure S15. Pesticide dissipation curves of spirotetramat (parent) in apples from 2020 (A) and 2021 (B) – later pesticide applications; from 2022 (C) and 2023 (D) – earlier pesticide applications.

Figure S16. Dissipation curves of spirotetramat and its metabolites in apple varieties Rosana (A) and Selena (B) in 2020.

Figure S17. Dissipation curves of spirotetramat and its metabolites in apple varieties Rosana (A) and Selena (B) in 2021.

Figure S18. Dissipation curves of spirotetramat and its metabolites in apple varieties Rosana (A) and Selena (B) in 2022.

Figure S19. Dissipation curves of flonicamid and its metabolites in apple varieties Rosana (A) and Selena (B) in 2020.

Figure S20. Dissipation curves of flonicamid and its metabolites in apple varieties Rosana (A) and Selena (B) in 2022.

TABLE CAPTIONS:

Table S1. The range of authorized uses of tested plant protection products for apple trees in the Czech Republic.

Table S2. The detailed timetable of pesticide treatments in a four-year field trial.

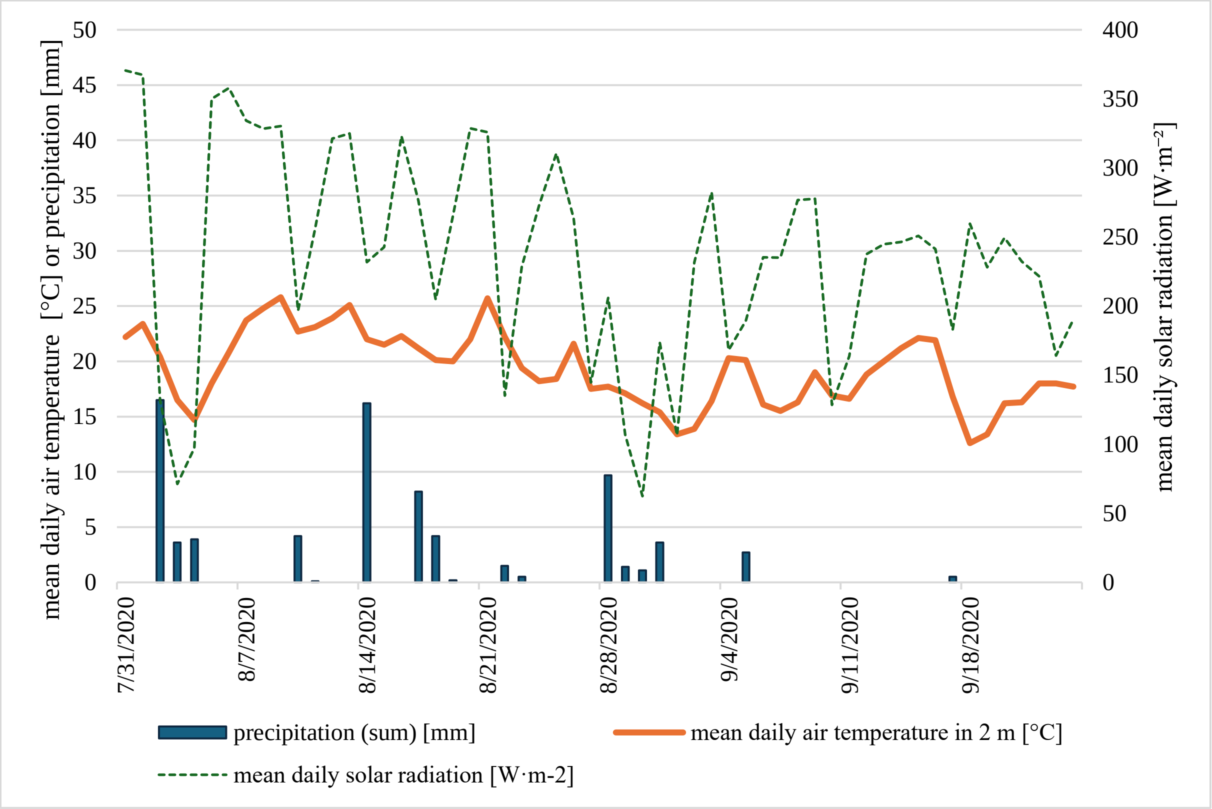

Figure S1.

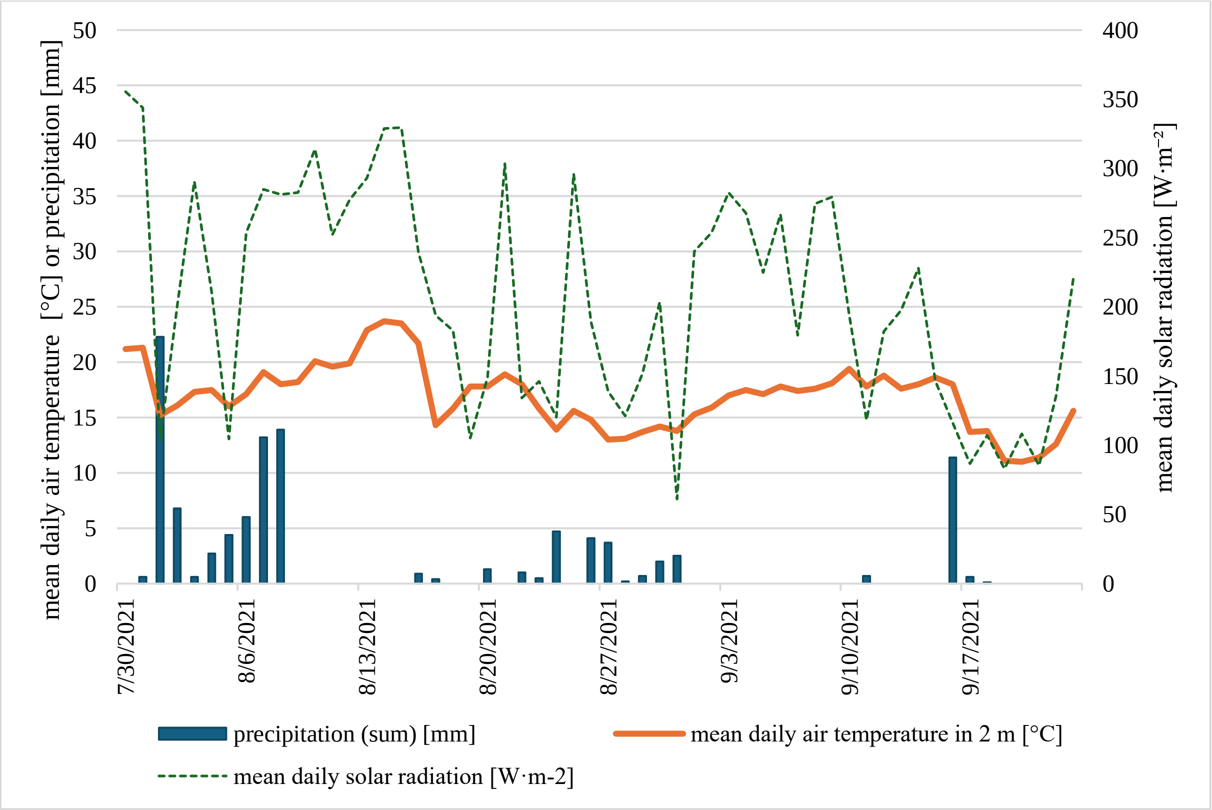

Figure S2.

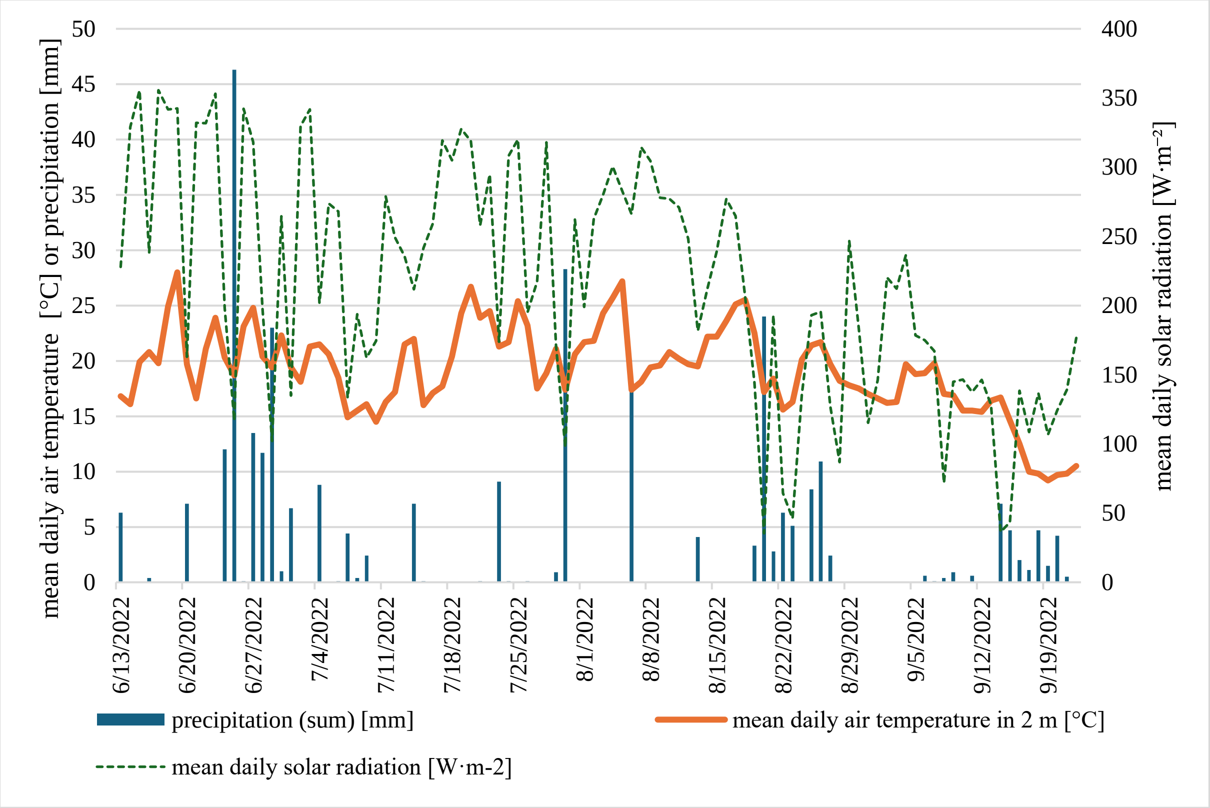

Figure S3.

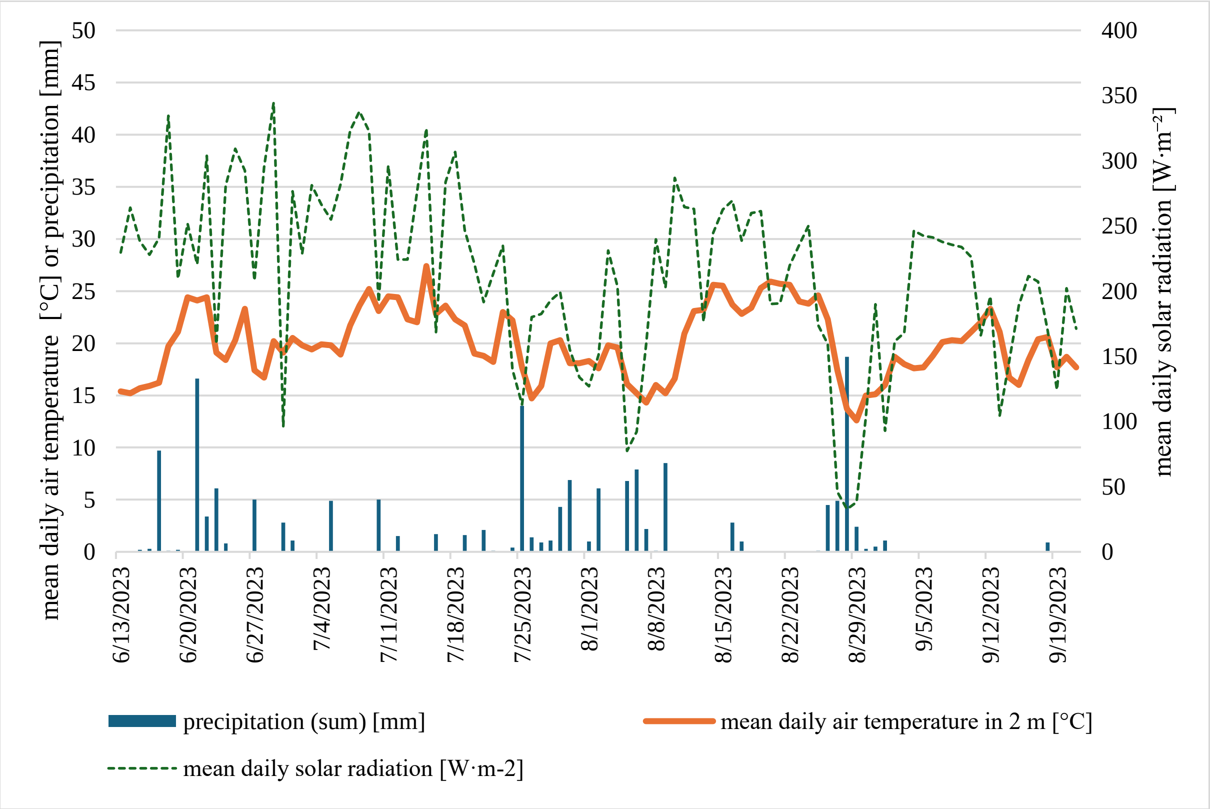

Figure S4.

Table S1.

trade name of PPP	manufacturer of PPP	active ingredient	pest/group of pests	number of applications/year
Exirel	FMC Agro Czech Republic Ltd.	cyantraniliprole	tortricid moths	1
Coragen 20 SC	FMC Agro Czech Republic Ltd.	chlorantraniliprole	tortricid moths, codling moth	1
Harpun	Galenika-Fitofarmacija A.D.	pyriproxyfen	codling moth (eggs)	2
Mimic	Nisso Chemical Europe GmbH	tebufenozide	codling moth	1
Mospilan 20 SP	Nisso Chemical Europe GmbH	acetamiprid	codling moth, aphids, woolly apple aphid	1
Movento® 100 SC	Bayer AG	spirotetramat *	green apple aphid, rosy apple aphid, woolly apple aphid, armored scales, apple leaf curling midge	2
Pirimor 50 WG	Adama Ltd.	pirimicarb	aphids	2
Sivanto Prime	Bayer AG	flupyradifurone	aphids, apple sawfly, apple psyllid	1 / two years
SpinTor	Corteva Agriscience™	spinosad	apple blossom weevil, psyllid species, tortricid moths, codling moth, apple sawfly	2
Teppeki	ISK Biosciences Corporation	flonicamid	aphids	3

* Expiration of approval in EU 07/31/2024

Reference:

Central Institute for Supervising and Testing in Agriculture. 2025. "Rostlinolékařský Portál." https://eagri.cz/public/app/srs_pub/fytoportal/fy-public/?k=0#rlp|domu|uvod. (Accessed 1/2/2025)

Commission Implementing Regulation (EU) 2022/489 of 25 March 2022 amending Implementing Regulation (EU) No 540/2011 as regards the approval periods of the active substances flubendiamide, L-ascorbic acid, spinetoram and spirotetramat; *OJ L 100, 28.3.2022, p. 7–9.*

Table S2.

**year of trial**	**2020**	**2021**	**2022**	**2023**
group I: date of treatment	07/31	07/30	06/13	06/13
group II: date of treatment	08/11	08/10	06/30	06/26
acetamiprid (Mospilan 20 SP)	group I	group II	group I	group II
chlorantraniliprole (Coragen 20 SC)	group II	group II	group I	group II
cyantraniliprole (Benevia/Exirel)	×*	group I	group II	group I
flonicamid (Teppeki)	group II	×	group II	group II
flupyradifurone (Sivanto Prime)	×	group II	group II	group I
pirimicarb (Pirimor 50 WG)	group II	group II	group I	group II
pyriproxyfen (Harpun)	group I	group II	group I	group I
spinosad (SpinTor)	group I	group II	×	×
spirotetramat (Movento 100 SC)	group II	group II	group I	group II
tebufenozide (Mimic)	×	×	group II	group I

* not applied in the experimental apple orchard

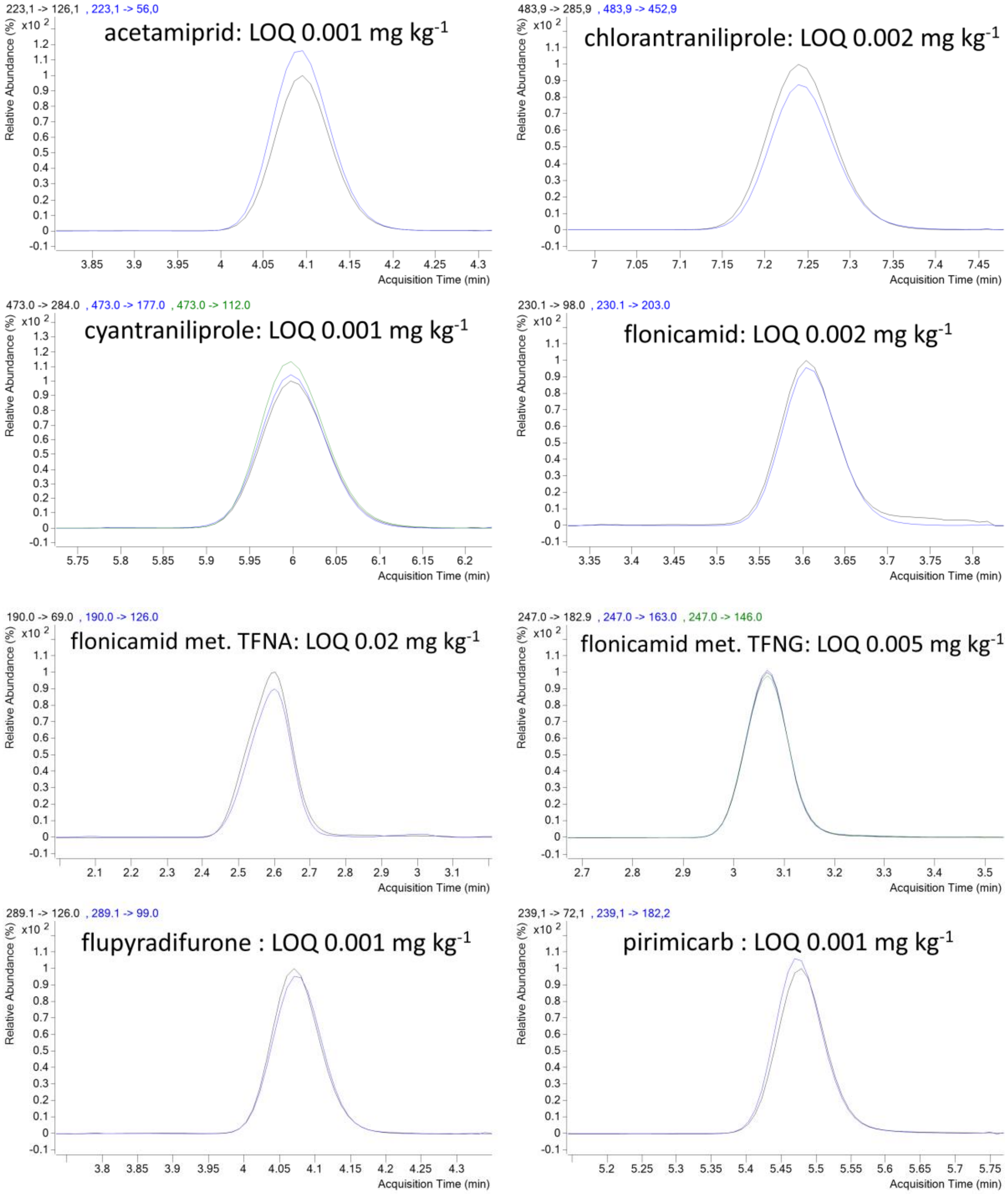
223,1 -> 126,1 , 223,1 -> 56,0
acetamiprid: LOQ 0.001 mg kg-1
483,9 -> 285,9 , 483,9 -> 452,9
chlorantraniliprole: LOQ 0.002 mg kg-1
473.0 -> 284.0 , 473.0 -> 177.0 , 473.0 -> 112.0
cyantraniliprole: LOQ 0.001 mg kg-1
230.1 -> 98.0 , 230.1 -> 203.0
flonicamid: LOQ 0.002 mg kg-1
190.0 -> 69.0 , 190.0 -> 126.0
flonicamid met. TFNA: LOQ 0.02 mg kg-1
247.0 -> 182.9 , 247.0 -> 163.0 , 247.0 -> 146.0
flonicamid met. TFNG: LOQ 0.005 mg kg-1
289.1 -> 126.0 , 289.1 -> 99.0
flupyradifurone : LOQ 0.001 mg kg-1
239,1 -> 72,1 , 239,1 -> 182,2
pirimicarb : LOQ 0.001 mg kg-1
x10 2
Relative Abundance (%)
Acquisition Time (min)

Figure S5.

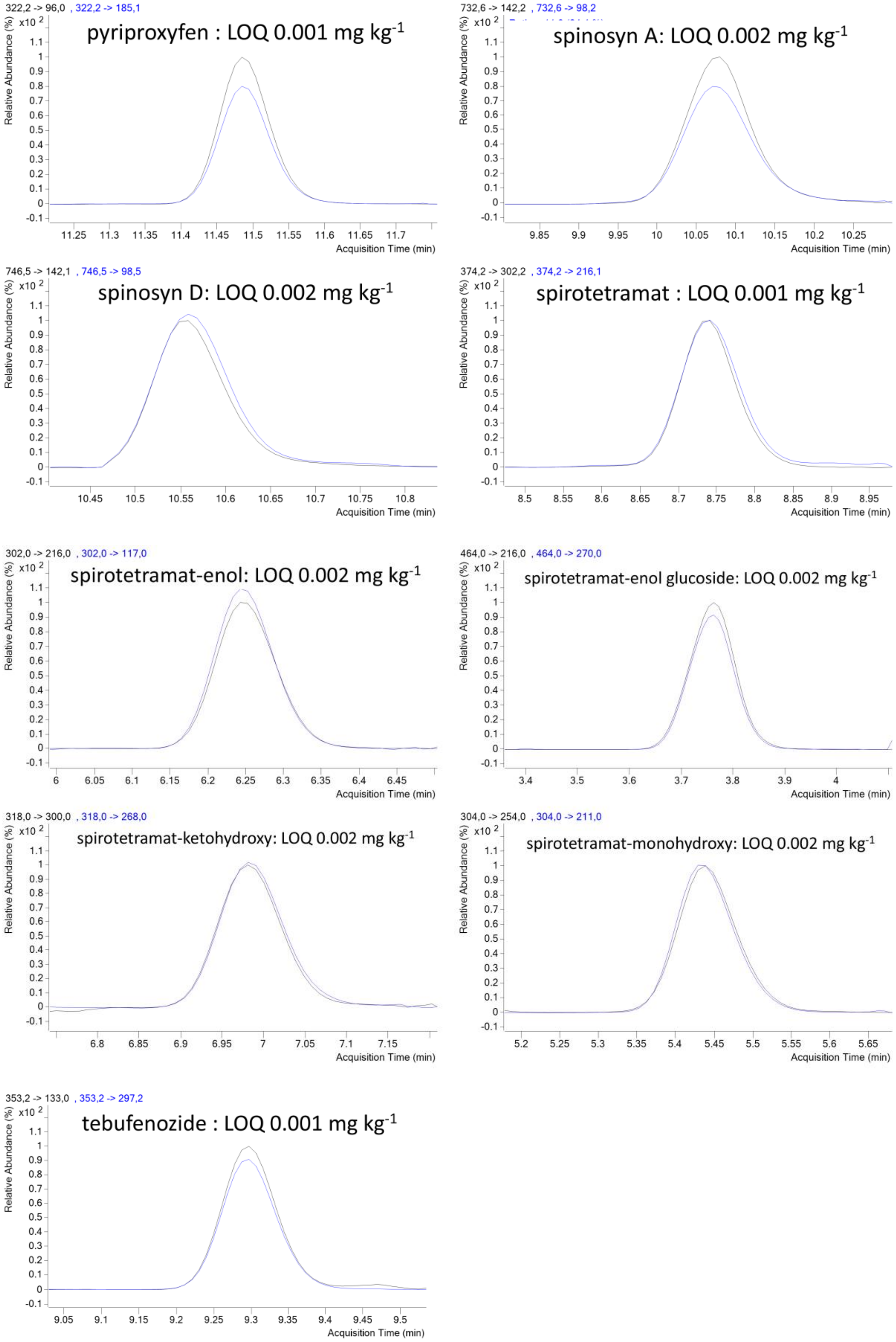

Figure S5. (continued)

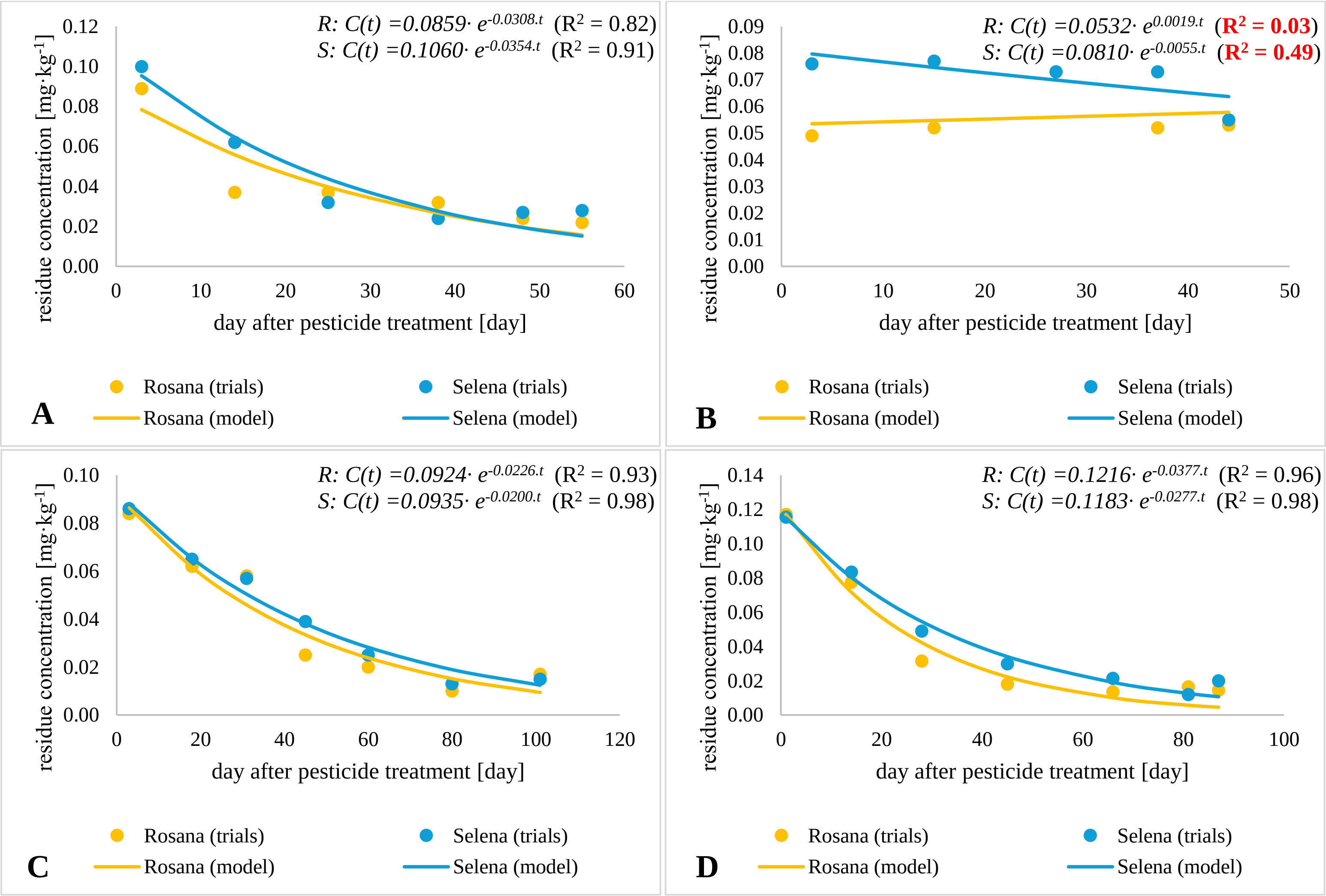

R: C(t) =0.0859· e-0.0308.t (R2 = 0.82)
S: C(t) =0.1060· e-0.0354.t (R2 = 0.91)
residue concentration [mg·kg-1]
day after pesticide treatment [day]
Rosana (trials)
Selena (trials)
Rosana (model)
Selena (model)
A
R: C(t) =0.0532· e0.0019.t (R2 = 0.03)
S: C(t) =0.0810· e-0.0055.t (R2 = 0.49)
B
R: C(t) =0.0924· e-0.0226.t (R2 = 0.93)
S: C(t) =0.0935· e-0.0200.t (R2 = 0.98)
C
R: C(t) =0.1216· e-0.0377.t (R2 = 0.96)
S: C(t) =0.1183· e-0.0277.t (R2 = 0.98)
D

Figure S6.

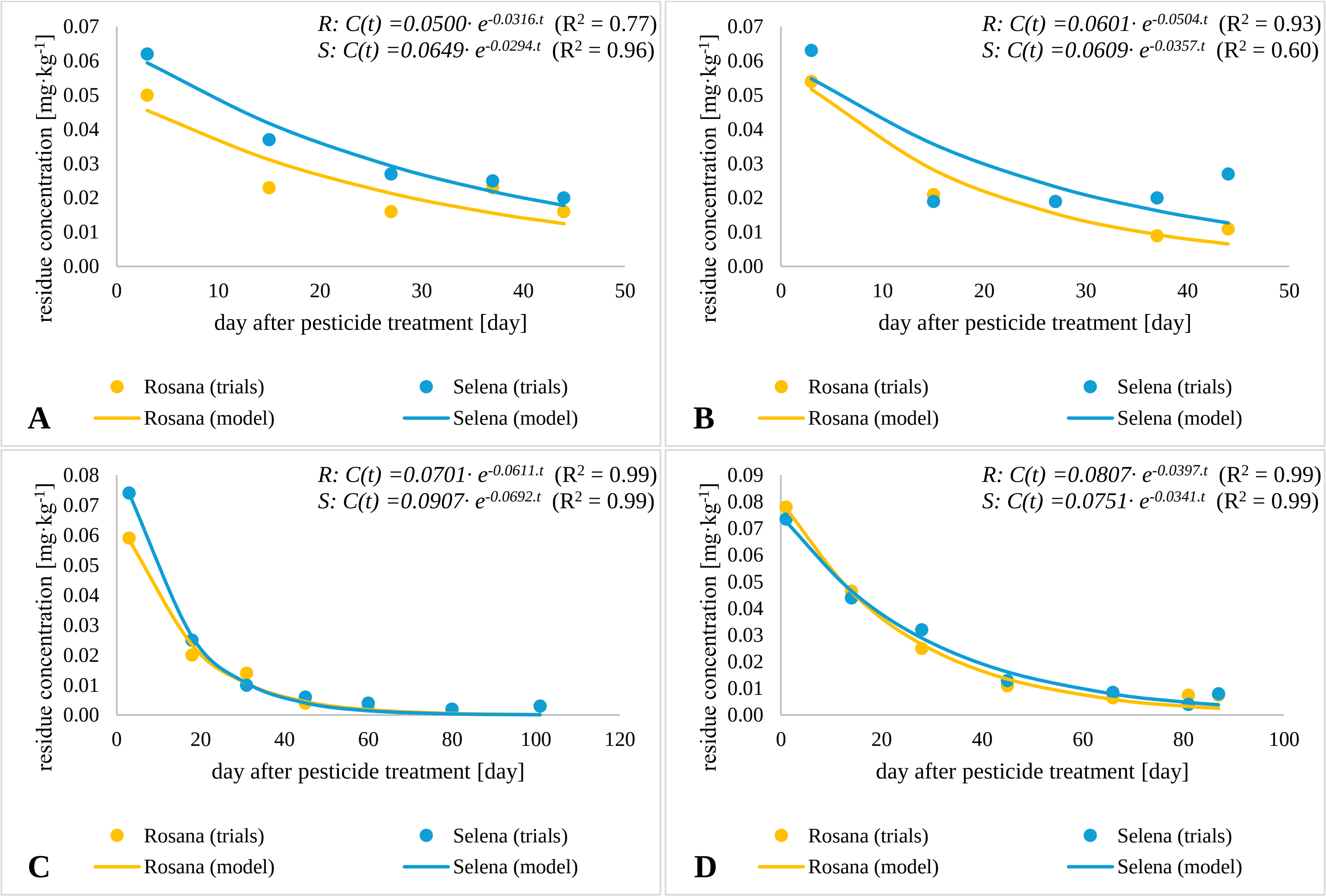

R: C(t) =0.0500· e-0.0316.t (R2 = 0.77)
S: C(t) =0.0649· e-0.0294.t (R2 = 0.96)
residue concentration [mg·kg-1]
day after pesticide treatment [day]
Rosana (trials)
Selena (trials)
Rosana (model)
Selena (model)
A
R: C(t) =0.0601· e-0.0504.t (R2 = 0.93)
S: C(t) =0.0609· e-0.0357.t (R2 = 0.60)
B
R: C(t) =0.0701· e-0.0611.t (R2 = 0.99)
S: C(t) =0.0907· e-0.0692.t (R2 = 0.99)
C
R: C(t) =0.0807· e-0.0397.t (R2 = 0.99)
S: C(t) =0.0751· e-0.0341.t (R2 = 0.99)
D

Figure S7.

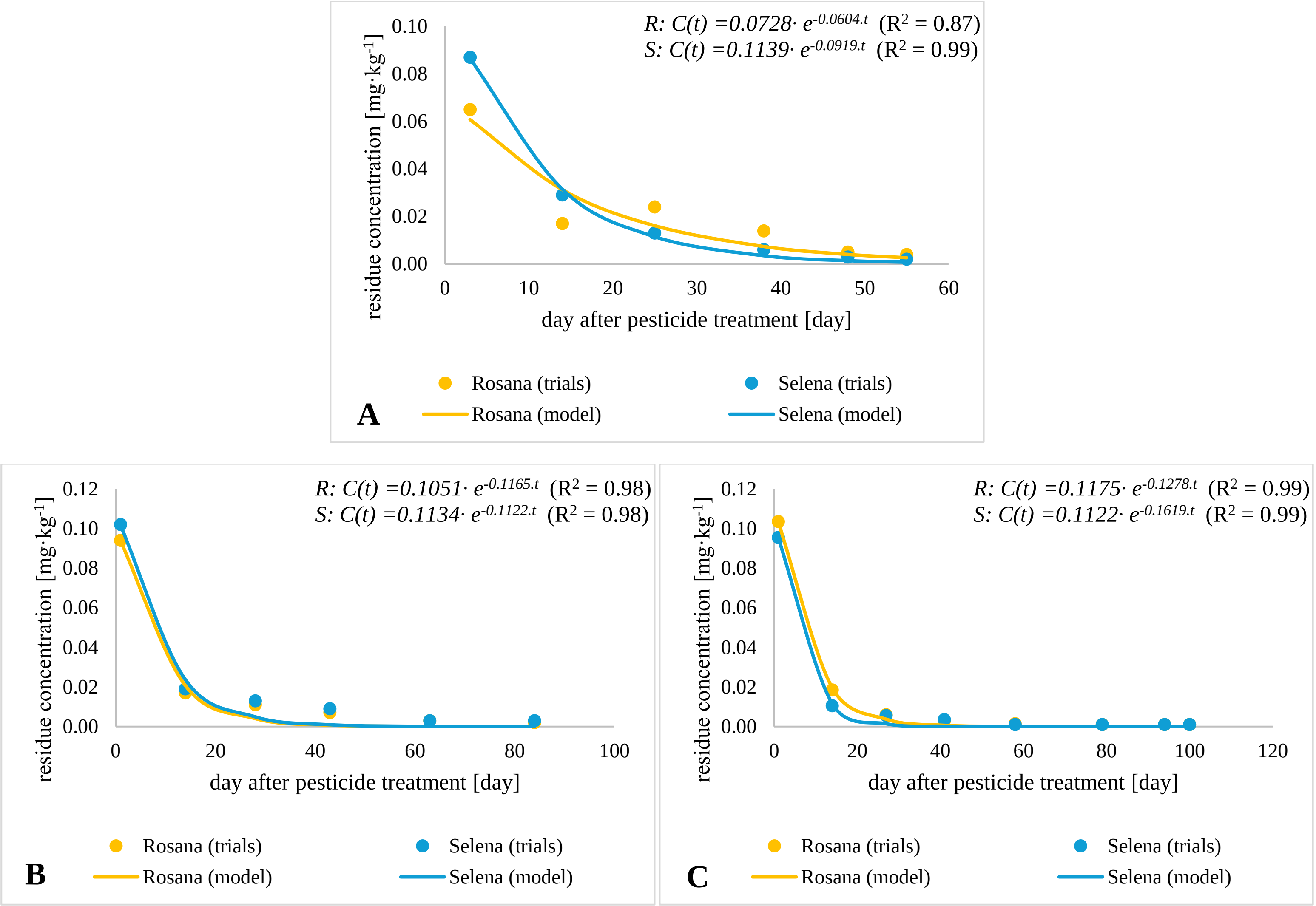
R: C(t) =0.0728· e-0.0604.t (R2 = 0.87)
S: C(t) =0.1139· e-0.0919.t (R2 = 0.99)
residue concentration [mg·kg-1]
0.10
0.08
0.06
0.04
0.02
0.00
0
10
20
30
40
50
60
day after pesticide treatment [day]
Rosana (trials)
Selena (trials)
A
Rosana (model)
Selena (model)
R: C(t) =0.1051· e-0.1165.t (R2 = 0.98)
S: C(t) =0.1134· e-0.1122.t (R2 = 0.98)
residue concentration [mg·kg-1]
0.12
0.10
0.08
0.06
0.04
0.02
0.00
0
20
40
60
80
100
day after pesticide treatment [day]
Rosana (trials)
Selena (trials)
B
Rosana (model)
Selena (model)
R: C(t) =0.1175· e-0.1278.t (R2 = 0.99)
S: C(t) =0.1122· e-0.1619.t (R2 = 0.99)
residue concentration [mg·kg-1]
0.12
0.10
0.08
0.06
0.04
0.02
0.00
0
20
40
60
80
100
120
day after pesticide treatment [day]
Rosana (trials)
Selena (trials)
C
Rosana (model)
Selena (model)

Figure S8.

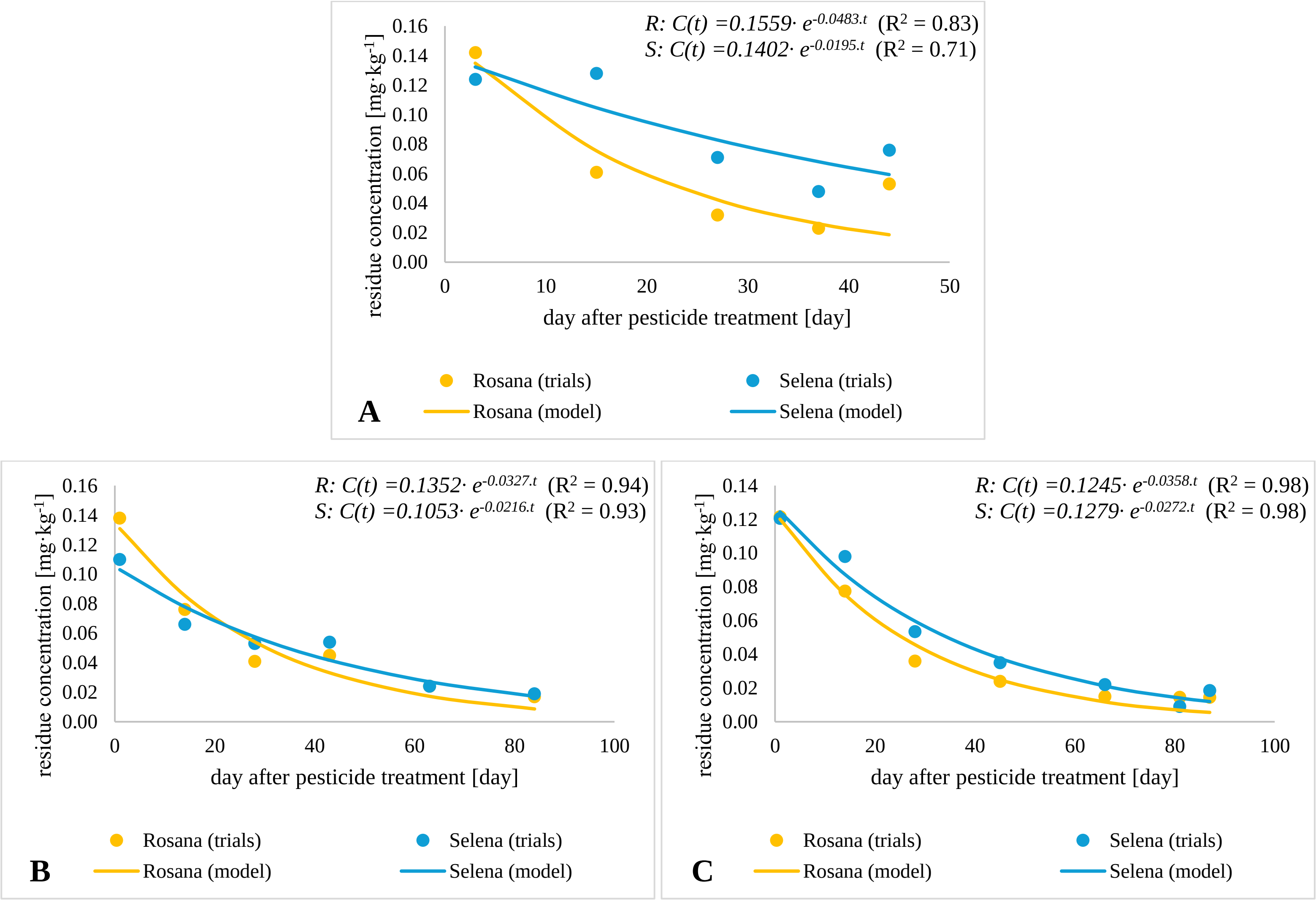

R: C(t) =0.1559· e^-0.0483.t (R² = 0.83)
S: C(t) =0.1402· e^-0.0195.t (R² = 0.71)
residue concentration [mg·kg-1]
day after pesticide treatment [day]
Rosana (trials)
Selena (trials)
Rosana (model)
Selena (model)
A
R: C(t) =0.1352· e^-0.0327.t (R² = 0.94)
S: C(t) =0.1053· e^-0.0216.t (R² = 0.93)
B
R: C(t) =0.1245· e^-0.0358.t (R² = 0.98)
S: C(t) =0.1279· e^-0.0272.t (R² = 0.98)
C

Figure S9.

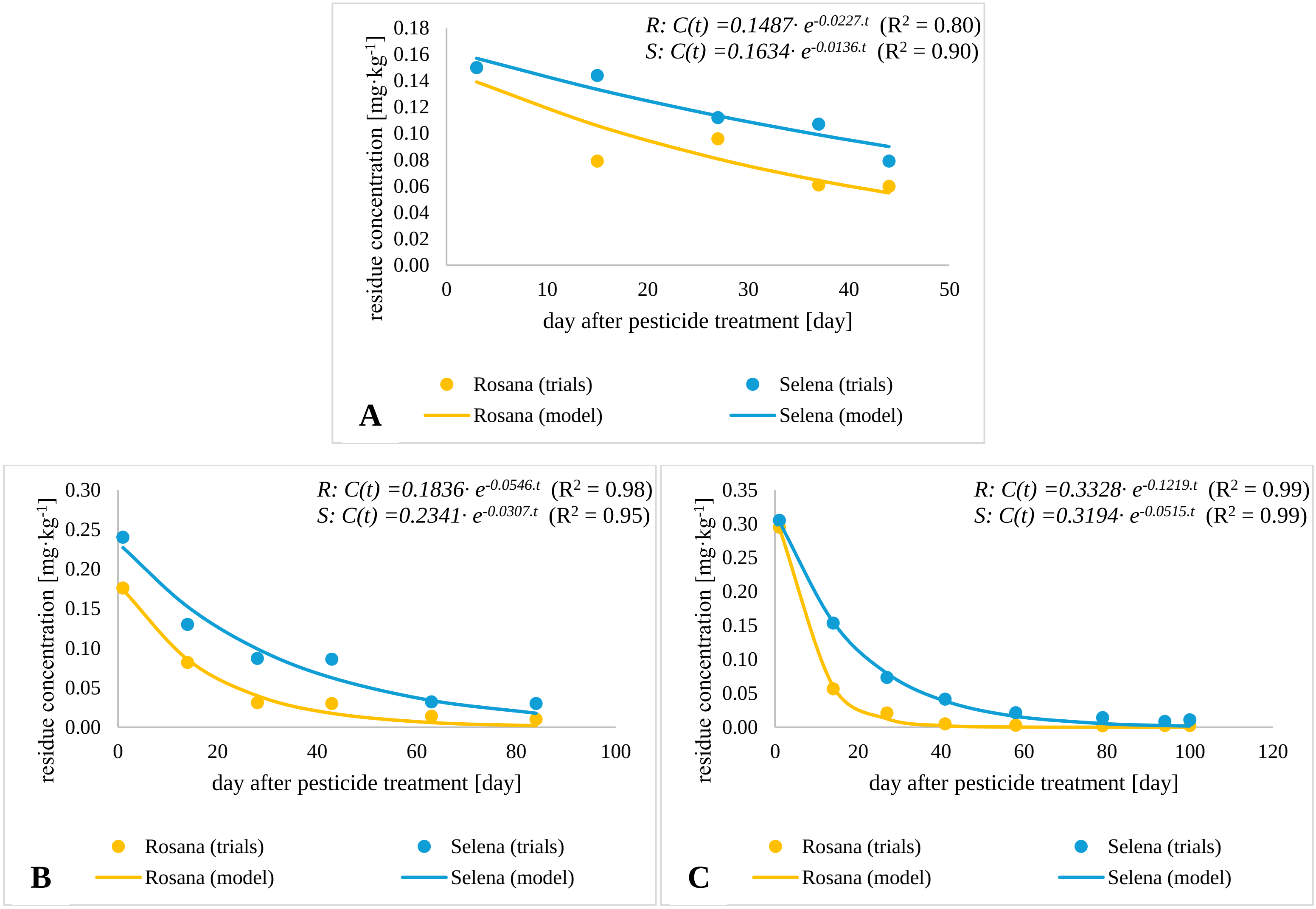

R: C(t) =0.1487· e-0.0227.t (R2 = 0.80)
S: C(t) =0.1634· e-0.0136.t (R2 = 0.90)
residue concentration [mg·kg-1]
day after pesticide treatment [day]
Rosana (trials)
Selena (trials)
Rosana (model)
Selena (model)
A
R: C(t) =0.1836· e-0.0546.t (R2 = 0.98)
S: C(t) =0.2341· e-0.0307.t (R2 = 0.95)
B
R: C(t) =0.3328· e-0.1219.t (R2 = 0.99)
S: C(t) =0.3194· e-0.0515.t (R2 = 0.99)
C

Figure S10.

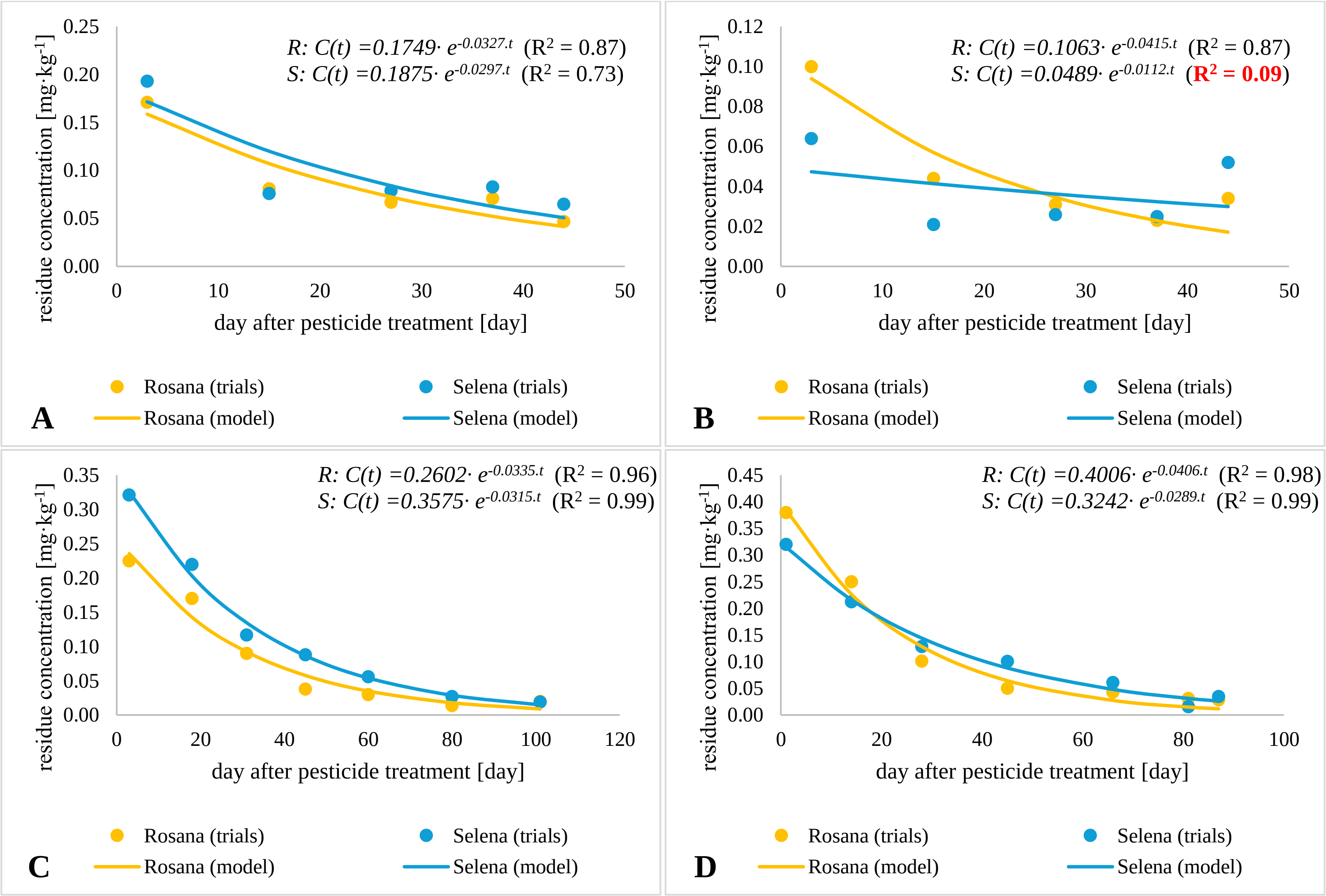

R: C(t) =0.1749· e-0.0327.t (R2 = 0.87)
S: C(t) =0.1875· e-0.0297.t (R2 = 0.73)
residue concentration [mg·kg-1]
day after pesticide treatment [day]
Rosana (trials)
Selena (trials)
Rosana (model)
Selena (model)
A
R: C(t) =0.1063· e-0.0415.t (R2 = 0.87)
S: C(t) =0.0489· e-0.0112.t (R2 = 0.09)
B
R: C(t) =0.2602· e-0.0335.t (R2 = 0.96)
S: C(t) =0.3575· e-0.0315.t (R2 = 0.99)
C
R: C(t) =0.4006· e-0.0406.t (R2 = 0.98)
S: C(t) =0.3242· e-0.0289.t (R2 = 0.99)
D

Figure S11.

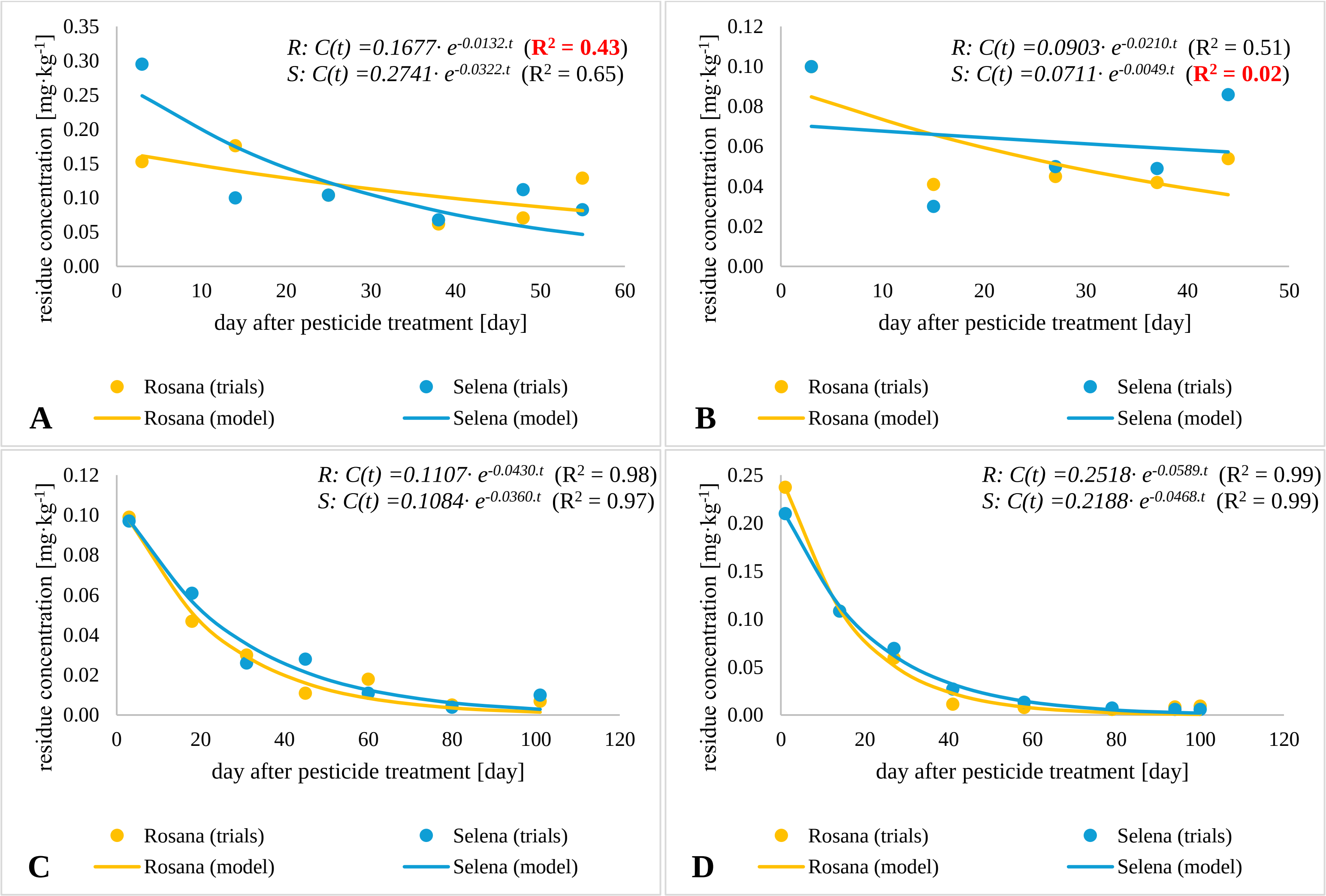

R: C(t) =0.1677· e-0.0132.t (R2 = 0.43)
S: C(t) =0.2741· e-0.0322.t (R2 = 0.65)
residue concentration [mg·kg-1]
day after pesticide treatment [day]
Rosana (trials)
Selena (trials)
Rosana (model)
Selena (model)
A
R: C(t) =0.0903· e-0.0210.t (R2 = 0.51)
S: C(t) =0.0711· e-0.0049.t (R2 = 0.02)
B
R: C(t) =0.1107· e-0.0430.t (R2 = 0.98)
S: C(t) =0.1084· e-0.0360.t (R2 = 0.97)
C
R: C(t) =0.2518· e-0.0589.t (R2 = 0.99)
S: C(t) =0.2188· e-0.0468.t (R2 = 0.99)
D

Figure S12.

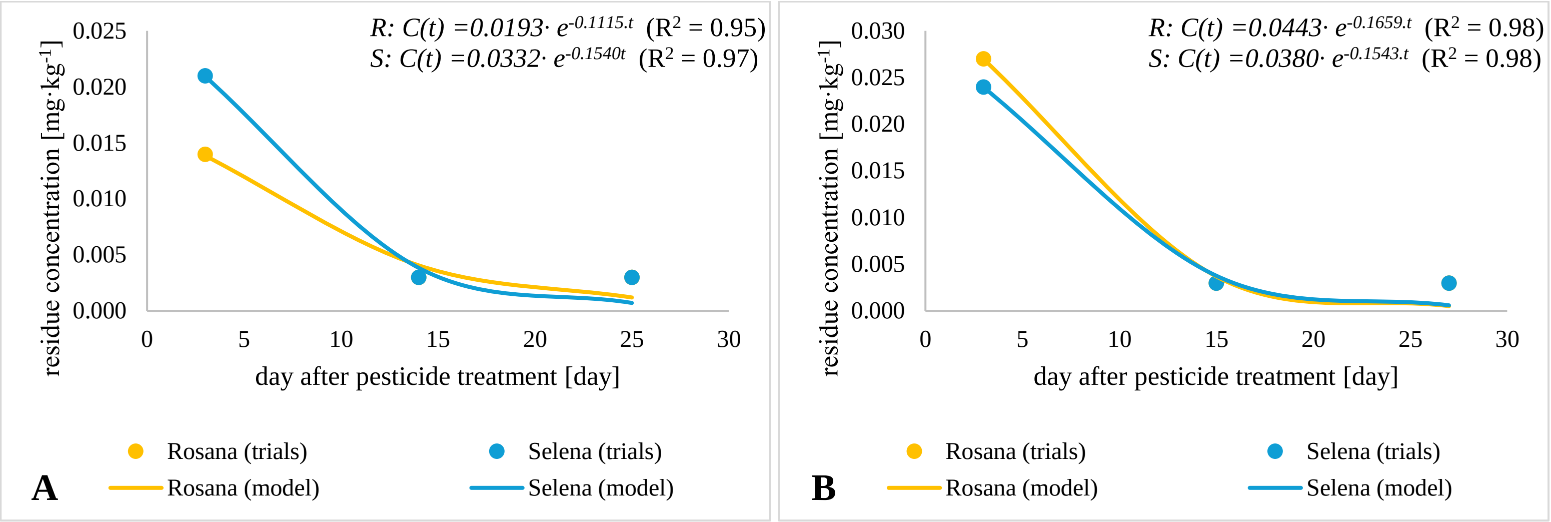

Figure S13.

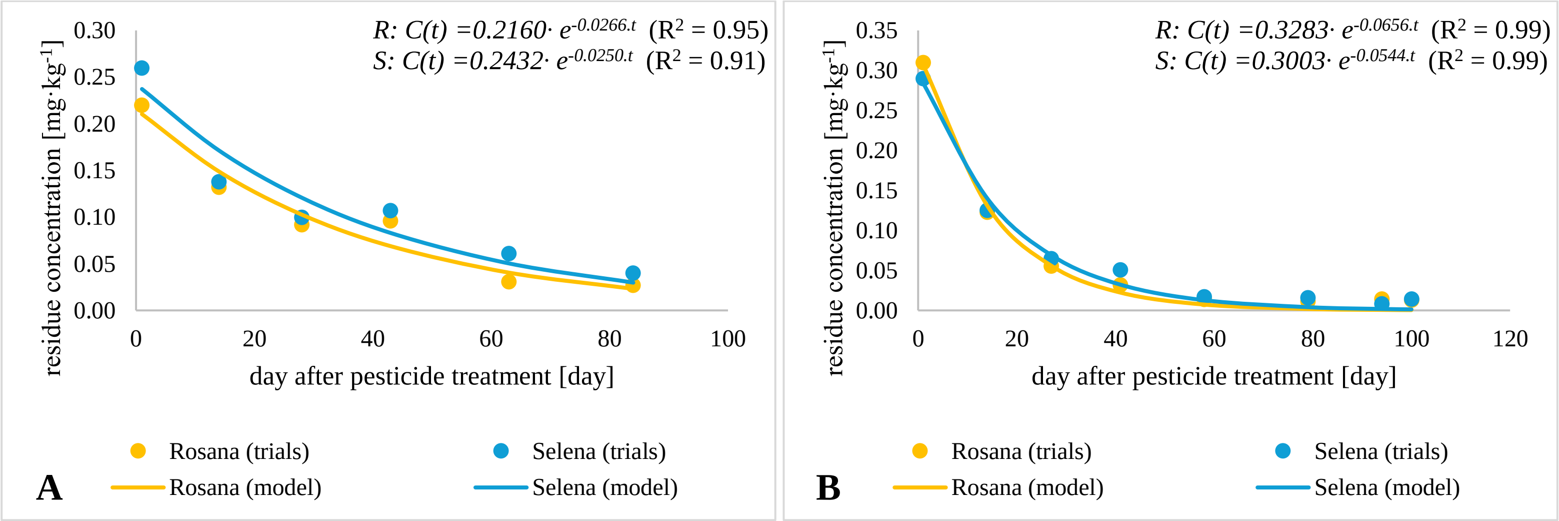

Figure S14.

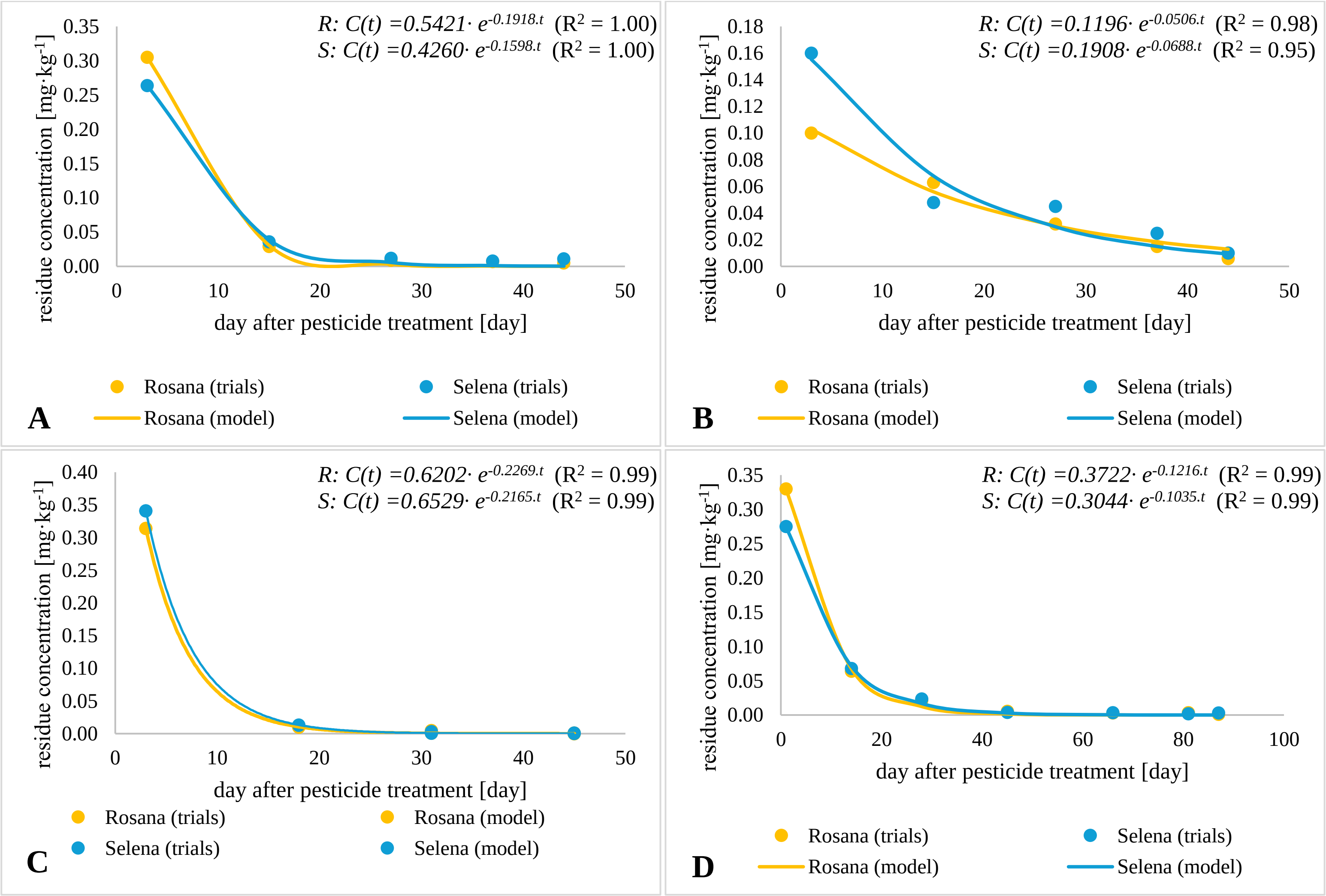
R: C(t) =0.5421· e-0.1918.t (R2 = 1.00)
S: C(t) =0.4260· e-0.1598.t (R2 = 1.00)
residue concentration [mg·kg-1]
day after pesticide treatment [day]
Rosana (trials)
Selena (trials)
Rosana (model)
Selena (model)
A
R: C(t) =0.1196· e-0.0506.t (R2 = 0.98)
S: C(t) =0.1908· e-0.0688.t (R2 = 0.95)
residue concentration [mg·kg-1]
day after pesticide treatment [day]
Rosana (trials)
Selena (trials)
Rosana (model)
Selena (model)
B
R: C(t) =0.6202· e-0.2269.t (R2 = 0.99)
S: C(t) =0.6529· e-0.2165.t (R2 = 0.99)
residue concentration [mg·kg-1]
day after pesticide treatment [day]
Rosana (trials)
Rosana (model)
Selena (trials)
Selena (model)
C
R: C(t) =0.3722· e-0.1216.t (R2 = 0.99)
S: C(t) =0.3044· e-0.1035.t (R2 = 0.99)
residue concentration [mg·kg-1]
day after pesticide treatment [day]
Rosana (trials)
Selena (trials)
Rosana (model)
Selena (model)
D

Figure S15.

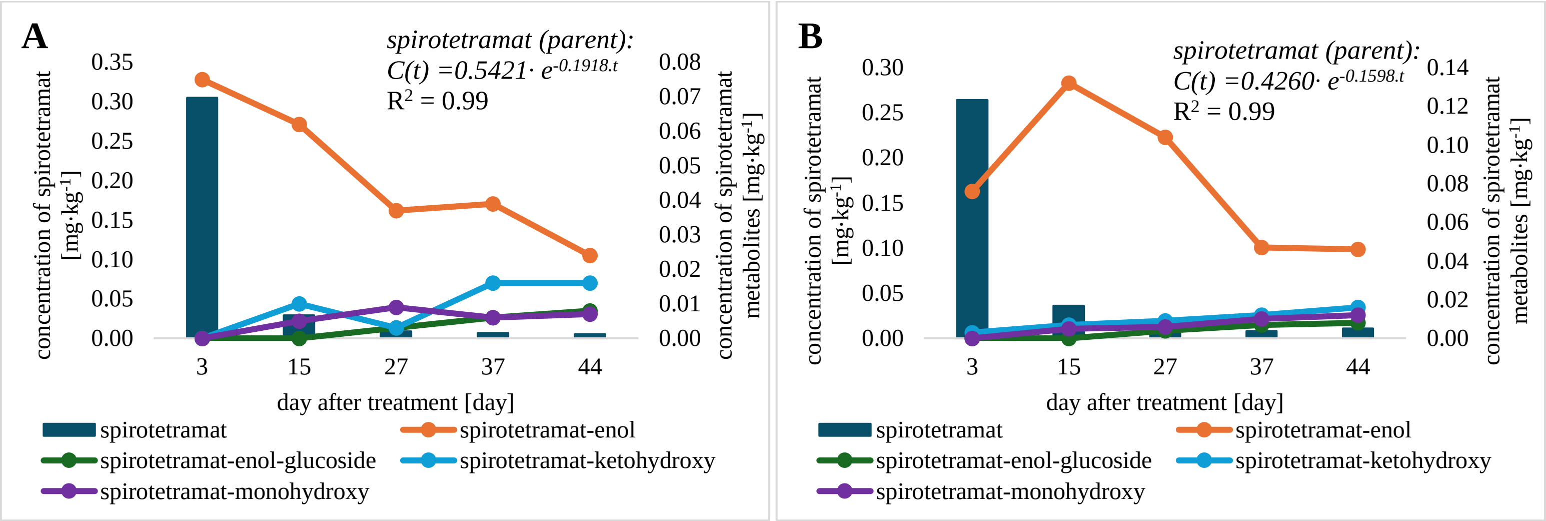

Figure S16.

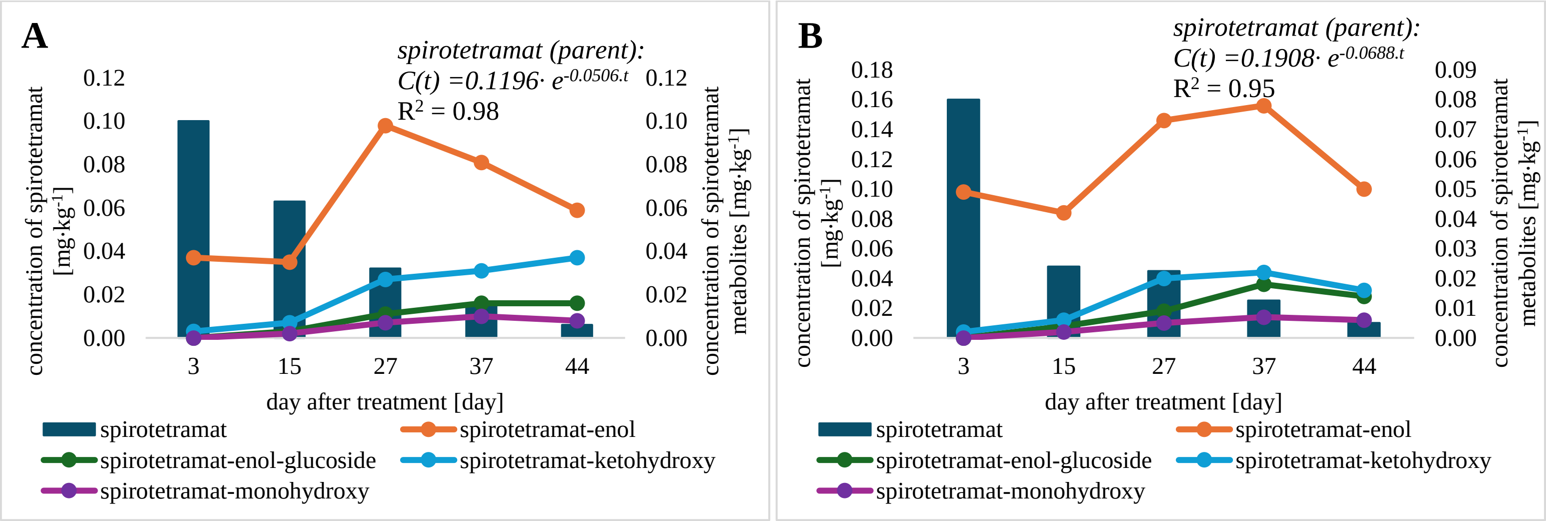

Figure S17.

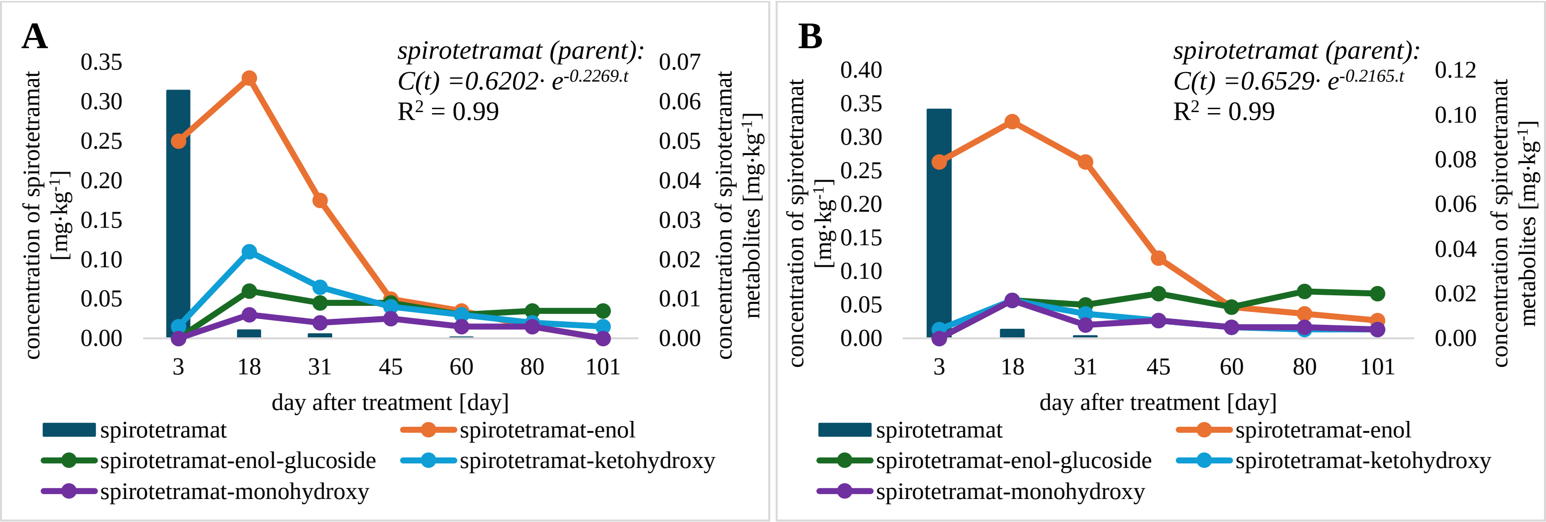

Figure S18.

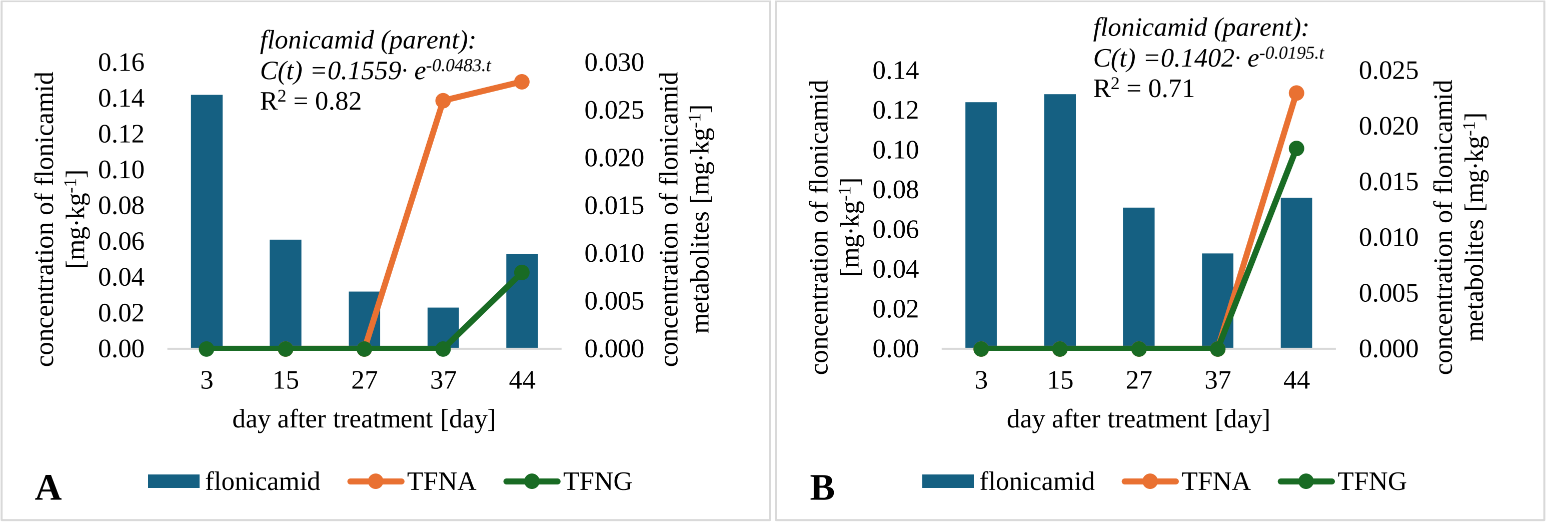

Figure S19.

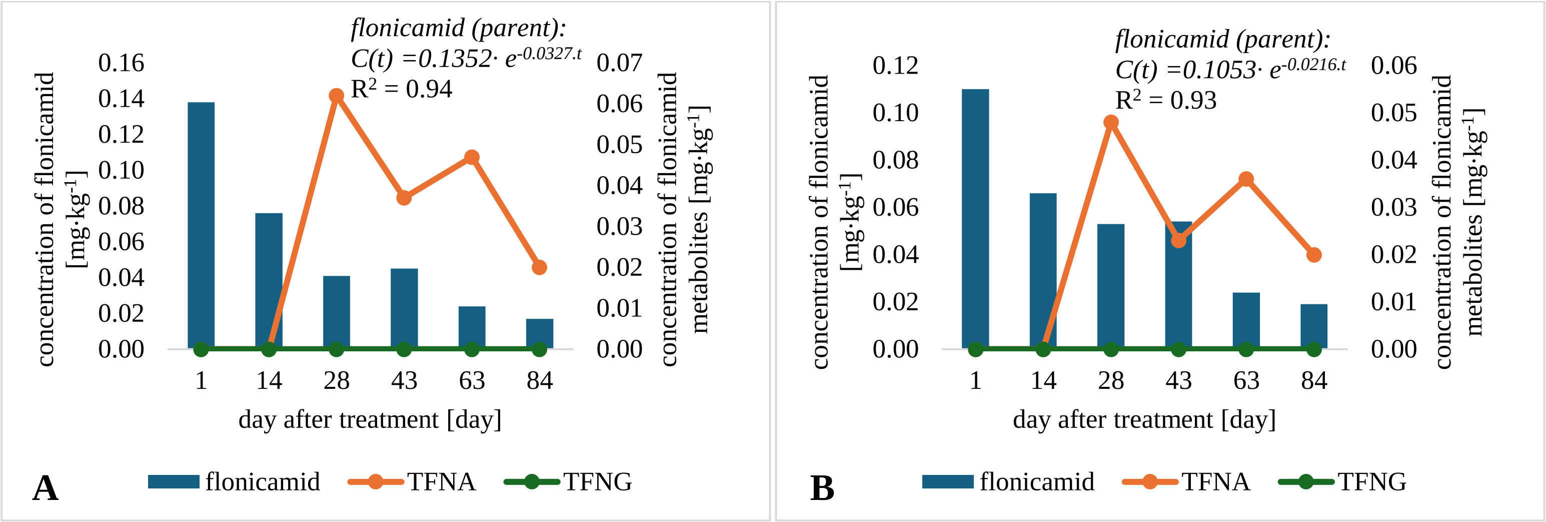

flonicamid (parent):
C(t) =0.1352· e-0.0327.t
R2 = 0.94
0.16
0.14
0.12
0.10
0.08
0.06
0.04
0.02
0.00
concentration of flonicamid [mg·kg-1]
0.07
0.06
0.05
0.04
0.03
0.02
0.01
0.00
concentration of flonicamid metabolites [mg·kg-1]
1
14
28
43
63
84
day after treatment [day]
A
flonicamid
TFNA
TFNG
flonicamid (parent):
C(t) =0.1053· e-0.0216.t
R2 = 0.93
0.12
0.10
0.08
0.06
0.04
0.02
0.00
concentration of flonicamid [mg·kg-1]
0.06
0.05
0.04
0.03
0.02
0.01
0.00
concentration of flonicamid metabolites [mg·kg-1]
1
14
28
43
63
84
day after treatment [day]
B
flonicamid
TFNA
TFNG

Figure S20.
